# The Development of Early Phonological Networks: An Analysis of Individual Longitudinal Vocabulary Growth

**DOI:** 10.1111/cogs.70109

**Published:** 2025-09-01

**Authors:** Judith Kalinowski, Laura Hansel, Michaela Vystrčilová, Alexander Ecker, Nivedita Mani

**Affiliations:** ^1^ Psychology of Language Research Group University of Göttingen; ^2^ Leibniz‐ScienceCampus Primate Cognition; ^3^ RTG 2636 Form‐meaning mismatches University of Göttingen; ^4^ Institute of Computer Science and Campus Institute Data Science University of Göttingen; ^5^ Max Planck Institute for Dynamics and Self‐Organization University of Göttingen

**Keywords:** Phonological networks, Longitudinal vocabulary development, Word learning, Phonological distance

## Abstract

While much work has emphasized the role of the environment in language learning, research equally reports consistent effects of the child's knowledge, in particular, the words known to individual children, in steering further lexical development. Much of this work is based on cross‐sectional data, assuming that the words typically known to children at *n* months predict the words typically known to children at *n*+x months. Given acknowledged variability in the number of words known to individual children at different ages, a more conclusive analysis of this issue requires examination of individual differences in the words learned by individual children across development, that is, using longitudinal data. In the current study, using longitudinal vocabulary data from children learning Norwegian, we ask whether the phonological connectivity of a word to words that the child already knows or words in the child's environment predicts the likelihood of the child learning that word across development. The results suggest that the early vocabulary grows predominantly in a rich‐get‐richer manner, where word learning is predicted by the connectivity of a word to already known words. However, word learning is, to a lesser extent, also influenced by the connectivity of a word to words in the child's linguistic environment. Our results highlight the promise of using longitudinal data to better understand the factors that influence vocabulary development.

## 1 Introduction

Recent approaches to language development take recourse to the characteristics of the environment in attempting to explain the pattern of language acquisition. For instance, with regard to lexical development, studies suggest that the number of words that the child is exposed to in their (caregiver) input predicts the child's expressive vocabulary development (Hart, 1991); the quality of speech directed to children, that is, whether speech is produced in an infant‐directed or child‐directed manner, predicts language processing and development (Outters, Schreiner, Behne, & Mani, 2020; Weisleder & Fernald, 2013); and infants' early babbling has been shown to adapt to their caregiver's productions (Goldstein & Schwade, 2008). The environment may also shape or trigger specific biases in children, such that exposure to certain objects or events in their input may lead to children following such biases in their subsequent language acquisition. For instance, children learn words easier when they know many other words that sound similar to (Newman, Samuelson, & Gupta, 2008) or overlap semantically with (Ackermann, Hepach, & Mani, 2020; Borovsky, Ellis, Evans, & Elman, 2016) the to‐be‐learned word and segment words from fluent speech easier when they sound similar to already‐known words (Altvater‐Mackensen & Mani, 2013). A similar trend is noticed in early productions, with early words being adapted to match previously familiar structures that the child can already produce well (Laing, 2019; Vihman, 2016; c.f. Ferguson & Farwell, 1975; Vihman, 2019). Thus, what children already know may shape the course of what children are likely to learn next, in interaction with the environment. Taken together, this body of work suggests that frequent and/or well‐rehearsed patterns may be recognized or produced more easily, due to infants' familiarity with these patterns. In subsequent learning, infants can then leverage such familiarity in learning new words that share features with these familiar words (Barabási & Albert, 1999; Mani & Ackermann, 2018). However, as we discuss next, accounts differ with regard to whether infants need to merely be exposed to these words in their input or need to already know these words, that is, understand the meaning of and/or produce these words.

Broadly speaking, the evidence briefly outlined above is consistent with prominent theories of lexical acquisition in early development. Thus, one set of theories, typically referred to as theories of preferential acquisition (Fourtassi, Bian, & Frank, 2020; Hills, Maouene, Maouene, Sheya, & Smith, 2009), focuses on the connectedness of words in the child's environment, with highly connected words sharing phonological or semantic features with many words in the child's environment and less connected words sharing features with fewer words in the child's environment. Theories of preferential acquisition suggest that words related to highly connected words in the child' environment are more likely to be learned next than words sharing features with less connected words in the child's environment. Following Fourtassi, Bian, and Frank (2020), we will refer to preferential acquisition as EXT to emphasize the external role of the input to the child in this growth scenario. Theories of preferential attachment, on the other hand, suggest that children are more likely to learn words that are similar to words they already know that are highly connected in their vocabulary networks (Barabási & Albert, 1999; Steyvers & Tenenbaum, 2005). Again, in keeping with Fourtassi, Bian, and Frank (2020), we will refer to preferential attachment as INT to highlight the internal role of the words known to the child in this growth scenario. A number of studies have examined the psychological reality of the above suggestions by examining vocabulary network growth in data from children at different ages. In particular, these studies have examined whether the (predominantly) semantic connectivity of words that the child already knows (preferential attachment, INT) or words in the child's environment (preferential acquisition, EXT) predict how likely a word is to be learned by the child.^1^

As we detail below, some of these studies find evidence for INT, while others find evidence for EXT. There are, however, a number of critical differences between these studies that call for further investigation of this issue. In particular, almost all studies use cross‐sectional data rather than longitudinal data of the words that individual children acquire. However, there are differences in the individual words known to individual children across development (Ackermann, Hepach, & Mani, 2020; Mani & Ackermann, 2018). If we want to examine how vocabulary growth is influenced by the words known to children, it is imperative that we look at the words actually known to individual children rather than relying on normative vocabulary data for the average child. Thus, there is a need for longitudinal investigation of the extent to which INT and EXT shape growth in the vocabularies of individual children (see Fourtassi, Bian, and Frank, 2020; Hills, Maouene, Maouene, Sheya, and Smith, 2009; Siew & Vitevitch, 2020b). Against this background, the current study will examine how INT and EXT shape vocabulary growth using data of the individual words known to individual children in a large longitudinal dataset of the words known to Norwegian children from 16 to 36 months of age.

Furthermore, most of the studies to‐date—aside from three notable exceptions described in detail below—have examined how the semantic connectivity between words shapes lexical development. Given the wealth of evidence documenting the influence of phonological connectivity on individual word learning (reviewed below), there is a need to examine how phonological connectivity in the vocabulary network shapes the pattern of early vocabulary growth. Against this background, the current study examines how phonological connectivity between words in individual children's lexicons and the environment shapes vocabulary growth using longitudinal data of children's vocabulary development.

In what follows, we first provide a brief introduction to the concept of vocabulary networks before describing the pattern of vocabulary growth proposed by preferential attachment and preferential acquisition (henceforth, referred to as INT and EXT vocabulary growth, respectively), followed by a review of studies examining this issue and the different measures of phonological distance they employed.

### 1.1 Vocabulary networks

**Fig. 1:**
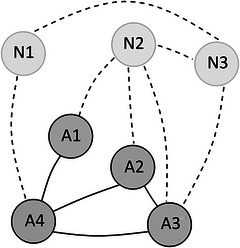
Example for a simple network.

Vocabularies are often represented as networks, where individual words, typically represented as the nodes of the network, are connected to one another, via the edges of the network, based on different features of overlap or similarity between these words. Network growth analysis has recently been used to investigate the factors influencing the pattern of vocabulary growth in young infants and children (e.g., Fourtassi, Bian, and Frank, 2020; Hills, Maouene, Maouene, Sheya, and Smith, 2009; Laing, 2025). Fig. 1, ^2^ shows an example for a simple network. The circles are the nodes or the elements of the network and the lines between them are the connections between these elements. When we represent a child's vocabulary and the words in its environment as a network, each element in the network represents a word. In Fig. 1, words A1–A4 are already‐learned words and thus represent the lexicon of a child. Words N1–N3 are yet‐to‐be acquired and would be new additions to the lexicon of the child. When we investigate phonological networks, the connections between words highlight that the words share a critical degree of phonological overlap. In other words, similar sounding words are connected with each other. In Fig. 1, word A4 is phonologically similar to words A1–A3 and N1, but not to N2 or N3.

Networks are described using certain key dimensions. Network density measures the proportion of actual connections to possible connections, with denser networks having a greater proportion of connections between words relative to sparser networks. The average path length of a network indicates the average number of steps needed to connect any two nodes within the network. The biggest subset of nodes where there is a path between any two nodes is typically referred to as the largest connected component of the network. This does not have to be a direct connection between two nodes; it can include intermediate connections, such as when node A is connected to node B and node B is connected to C, forming a path between word A and C. The number of hermits indicates how many nodes are not connected to any other node in the network. Finally, the greatest distance between any two nodes when considering the shortest path between each pair of nodes is referred to as network diameter.

### 1.2 INT vocabulary growth scenario

There is a large body of work suggesting that the words that children already know influence how children process and learn other related words (see (Mani & Ackermann, 2018) for a review). This work finds that children learn words that semantically overlap with (many) words they already know (and are interested in) easier than words that overlap with fewer words (Ackermann, Hepach, & Mani, 2020; Borovsky, Ellis, Evans, & Elman, 2016). Altvater‐Mackensen and Mani (2013) find that children are able to segment words from the fluent speech stream easier when these words sound similar to word‐forms they are familiar with. Similarly, children recognize words more robustly if they overlap phonologically (Altvater‐Mackensen and Mani, 2013; Mani & Plunkett, 2011), semantically (Arias‐Trejo & Plunkett, 2010), or if their referents overlap visually (Bobb, Huettig, & Mani, 2016) with words that the child already knows. With particular regard to how phonological neighbors influence word learning, studies find that words with multiple phonological neighbors are easier to learn than words with fewer or no phonological neighbors (Newman, Samuelson, & Gupta, 2008; Storkel, 2004). Finally, there is a rich literature on early word productions, suggesting that children's first words typically overlap phonologically (McCune & Vihman, 2001) and that children' familiarity with producing certain structures influences their perception of such structures in the input (DePaolis, Vihman, & Keren‐Portnoy, 2011; Majorano, Vihman, & DePaolis, 2014).

**Fig. 2:**
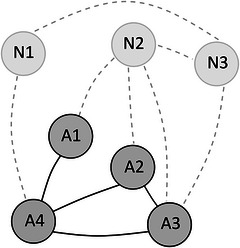
Visualization of the INT growth scenario. The edges between nodes $n_{i},n_{j}$ with $n_{i},n_{j}\in \left\{A1,\text{…},A4,N1,\text{…},N3\right\},n_{i}\neq n_{j}$ only exist if $dist(n_{i},n_{j})\leq 0.25$ (note that the distance metrics are defined in Section 1.5). The table shows the calculation of the INT values. The word with the highest value is highlighted in gray and hypothesized to be learned next.

The studies briefly reviewed above are in keeping with theories suggesting that lexical development follows the power law distribution. Thus, these theories propose that children learn words that are similar to words they already know that are highly connected in their vocabulary networks, that is, are themselves similar to many other words already known to the child (INT vocabulary growth scenario, Fourtassi, Bian, and Frank, 2020, otherwise known as preferential attachment, Barabási & Albert, 1999, Steyvers & Tenenbaum, 2005). We illustrate this using an example network in Fig. 2. As noted above, words A1–A4 are already‐learned words and thus represent the lexicon of a child. Words N1–N3 are yet‐to‐be acquired and would be new additions to the lexicon of the child. Were N1 to be learned, this word would connect to A4 in the existing network, which is connected to three other already‐learned words. Thus, N1 would have an INT value of 3, representing the connectivity of words N1 is similar to. N3 would be connected to one word, namely, A3, which is connected to 2 already‐learned words. Therefore, the INT value of N3 is 2. N2, however, would be connected to three different words, A1, A2, and A3. A1 is connected to A4, while A2 and A3 are each connected to two other words (each other and A4). As the INT‐value of a new word is the mean value of all connected words, we need to take the mean value of A1, A2, and A3 to get the INT‐value of N2, which is 1.7 (see Fig. 2 for the calculation). Given that the INT‐vocabulary growth scenario suggests that the word with the highest INT‐value will be acquired next, in our example, this would be max(INT(N1),INT(N2),INT(N3))=max(3,1.7,2)=3, that is, the word N1 would be learned next in an INT‐vocabulary growth scenario.

### 1.3 EXT vocabulary growth scenario

At the same time, much prior research suggests that the statistical properties of the child's environment play an important role in learning (Hart, 1991; Saffran, Aslin, & Newport, 1996; Smith & Yu, 2008). Thus, the frequency with which children hear particular words and how often words co‐occur with objects in their environments predicts how easily children learn words and their meanings (Saffran, Aslin, & Newport, 1996; Smith & Yu, 2008). Similarly, infants' segmentation of words from fluent speech is influenced by the quality of speech directed to them (Outters, Schreiner, Behne, & Mani, 2020; Weisleder & Fernald, 2013). In terms of early productions, studies suggest that the number of words that the child is exposed to in their input predicts the child's expressive vocabulary development (Hart, 1991). Even the properties of infants' babbling appears to be shaped by their input, with infant babbling adapting to auditory prompts provided by their caregivers, that is, aligning with the phonological form of their caregiver's productions (Goldstein & Schwade, 2008). Given this powerful role of the input in driving early learning, another set of theories suggests that words that sound similar to highly connected words in the child's learning environment are learned easier (EXT vocabulary growth scenario (Fourtassi, Bian, and Frank, 2020), preferential acquisition in semantic networks (Hills, Maouene, Maouene, Sheya, and Smith, 2009)), that is, regardless of whether the words in the environment are already known to the child or not.

**Fig. 3:**
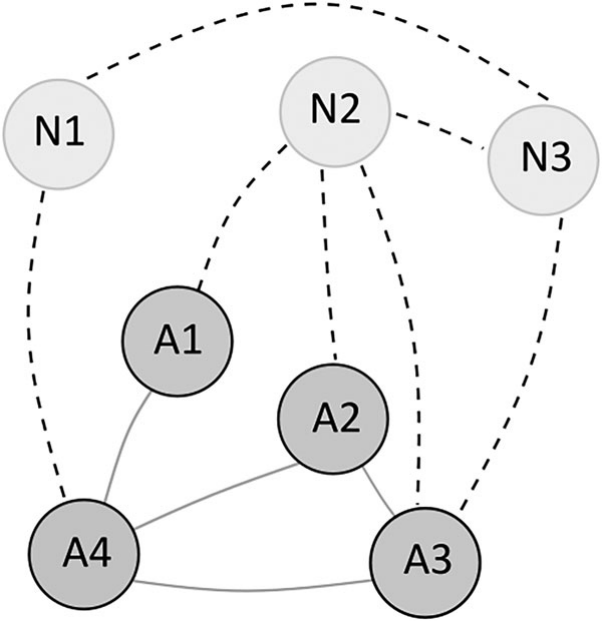
Visualization of the EXT growth scenario. The edges between nodes $n_{i},n_{j}$ with $n_{i},n_{j}\in \left\{A1,\text{…},A4,N1,\text{…},N3\right\},n_{i}\neq n_{j}$ only exist if $dist(n_{i},n_{j})\leq 0.25$ (note that the distance metrics are defined in Section 1.5). The table shows the calculation of the EXT values. The word with the highest value is highlighted in gray and hypothesized to be learned next.

Fig. 3 shows the same exemplary network as before. All words are in the environment of the child, and, therefore, all words influence the likelihood of a word to be learned, regardless of whether they are known to the child or not. N1 connects to both A4 (in the existing network) and N3 (in the end‐state network). Therefore, the EXT value of N1 would be 2. N2 has an EXT value of 4, because it connects to A1–A3 and N3. Finally, N3 has links to A3, N1, and N2, and its EXT value is, therefore, 3. In an EXT vocabulary growth scenario, the word that is connected to the most words in the child's environment would be learned next. Therefore, word N2 would, therefore, be learned next, because max(EXT(N1), EXT(N2), EXT(N3)) = max(2, 4, 3) = 4.

Estimates of the words in the child's environment are difficult to obtain. Therefore, the presence of words in the environment of the child is typically assumed based on their inclusion in vocabulary inventories—the so‐called end‐state network (Fourtassi, Bian, and Frank, 2020; Hills, Maouene, Maouene, Sheya, and Smith, 2009). In other words, vocabulary inventories typically include words that are likely to be learned by children at the upper end of the age spectrum for that inventory (the end‐state) and are, therefore, likely to be in the input to the child.

### 1.4 INT and EXT in past research

The role of INT or EXT vocabulary growth scenarios in phonological networks of children has been examined recently by Fourtassi, Bian, and Frank (2020), Laing (2025), Beckage and Colunga (2019), and Siew and Vitevitch (2020b). Surprisingly, the different studies came to different conclusions: Fourtassi, Bian, and Frank (2020) found evidence for EXT vocabulary growth in their analyses, while Laing (2025) found that phonological networks grow in an INT‐like manner. Neither of the studies found any evidence for the other growth scenario. Siew and Vitevitch (2020b) found evidence for both INT and EXT vocabulary growth scenarios in children between 3 and 9 years of age, while also finding that INT had a greater impact on the growth of phonological networks. Beckage and Colunga (2019) looked into longitudinal data from late talkers and suggest that an effect of INT on phonological and semantic network growth can only be found when we consider longitudinal data from individual children.

There are a number of potential reasons for the differing results across studies. First, the authors estimated the phonological similarity between words in different ways: Fourtassi, Bian, and Frank (2020) used Levenshtein distance, Laing (2025) used the phonetic distance between phonemes in the words, while Siew and Vitevitch (2020b) examined minimal pairs, that is, words were considered to be phonologically similar if they could be transformed to the other with the substitution, deletion, or addition of a single phoneme (Luce & Pisoni, 1998). We describe these methods in more detail in the Supplementary Materials. Second, there are key differences in the datasets entered into the analyses. Fourtassi, Bian, and Frank (2020) and Laing (2025) examined vocabulary growth at 1‐month intervals relative to the yearly intervals examined in Siew and Vitevitch (2020b). Fourtassi, Bian, and Frank (2020) used cross‐sectional CDI data from wordbank (Frank, Braginsky, Yurovsky, & Marchman, 2017), Laing (2025) used longitudinal data of (nine) children's productive vocabularies in two different languages (French/English). Siew and Vitevitch (2020b) used the Amazon Mechanical Turk platform and (Brysbaert, Stevens, De Deyne, Voorspoels, & Storms, 2014) as the basis for their analyses and looked at an older age group (3–9 years of age).

Importantly, Laing (2024) is the only study thus far to examine longitudinal data from typically developing children, albeit from only nine children. Indeed, most of the studies that have examined the role of these factors on lexical acquisition have used normative data, that is, based their conclusions on averaged data from many children. Thus, at each age, they approximated an average lexicon of words known to children at that age and examined the factors influencing the words acquired next, that is, at the next observation, also based on the average lexicon of words known to children at that age. Since different children learn and retain different words at different points across development (see Ackermann, Hepach, & Mani, 2020; Chi & Koeske, 1983; Rothwell, Westermann, & Hartley, 2023), a more appropriate test of the role of these factors on the pattern of lexical acquisition would be to examine longitudinal data (see Fourtassi, Bian, and Frank, 2020; Hills, Maouene, Maouene, Sheya, and Smith, 2009; Vitevitch, 2008 for similar arguments).

The current study will examine these issues bringing together the strengths of the literature thus far. In particular, we use longitudinal data, in order to examine individual vocabulary growth and how the phonological similarity of words known to individual children impacts the pattern of vocabulary growth. This allows us to estimate the influence of the words known to individual children on subsequent vocabulary development, relative to the predominantly normative work thus far. At the same time, in a key advance to the literature to date, we use data from publicly available vocabulary questionnaires to examine vocabulary growth in individual children. This not only gives us data from more children than can be analyzed using more time and resource‐intensive data of children's productions, but also raises the possibility of vocabulary norms being used in future research to answer similar questions. Finally, we examine the dynamics of the individual growth scenarios (Siew & Vitevitch, 2020a) by tracking them across development, that is, examining the extent to which the influence of INT or EXT vocabulary growth scenarios changes across development.

Note that we used different measures of the phonological similarity between words in order to compare the pattern of results obtained using such estimates in the literature thus far. In particular, we calculated the INT and EXT values three times, based on three different phonological similarity measurements (cf. Section 1.5). While we report the results using our main measure of phonological similarity, we note that we obtained very similar results (reported in the Supplementary Materials) using the other measures we examined. Furthermore, as in the studies reviewed above (Fourtassi, Bian, and Frank, 2020; Hills, Maouene, Maouene, Sheya, and Smith, 2009), we used the end‐state network as the proxy for the EXT values. While we could have chosen the last observation of each child as their final network, this led to considerable variability in the number of words in the final network, since the final observations were at different ages for different children. Since all children will know all words in the CDI at some point, we decided—in keeping with previous research—to take the whole CDI, the end‐state network as our final network. In what follows next, we describe the measure of phonological similarity used in the current study.

### 1.5 Phonological similarity

The phonological distance between words gives us a measure of how similar two words sound. The question of how best to capture the phonological similarity between words has been a matter of some debate for many years. Luce and Pisoni (1998), for example, discuss various ways of calculating the phonological similarity between words. However, the question is by no means easy to answer, as there are many dimensions of phonological similarity that need to be considered in calculating such metrics, including, for instance, how similar the sounds of two words are, the relevance of the onsets and ends of words (De Cara & Goswami, 2002; Marslen‐Wilson & Zwitserlood, 1989), and the relevance of consonants and vowels to word recognition (Mani & Plunkett, 2010; Nazzi, 2005; Nazzi & Polka, 2018), among others. This is especially so, since the different measures used may tap into different cognitive and linguistic aspects of phonological similarity and may be tailored to specific psycholinguistic questions (Castro & Vitevitch, 2023). Against this background, we included three different measures of phonological similarity in the current study that have been used in the literature to date. Since the results from the different measures did not differ considerably from one another, here, we focus on our main measure, which was an extended version of the phoneme feature distance by Monaghan, Christiansen, Farmer, and Fitneva (2010). The results with the other measures, including correlations between measures and our reasoning for focusing on the current measure, are presented in the Supplementary Materials. In what follows next, we present our main measure in more detail.

**Fig. 4:**
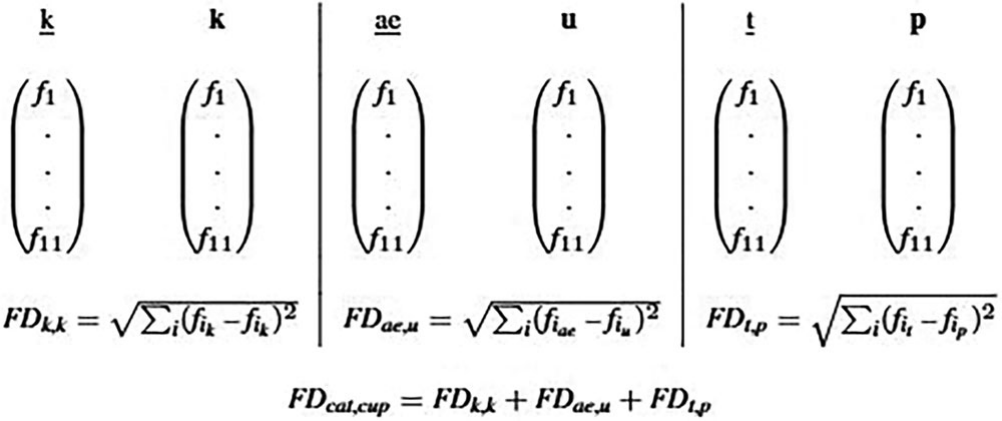
Example for the Euclidean distance of phoneme features based on FD. The phonemes of the words cat and **cup** next to each other with their 11 phonological features $f_{1},\text{…}f_{11},f_{i}\in \left\{-1,0,1\right\}$ (based on Harm & Seidenberg, 1999). The Euclidean distance between each phoneme pair is calculated.

Phoneme feature distance is calculated based on IPA transcriptions of the words to be compared. Phoneme feature distance, as proposed by Monaghan, Christiansen, Farmer, and Fitneva (2010), however, only considered mono‐syllabic words. We, therefore, adapted the computations for multi‐syllabic words, which we henceforth refer to as FDMS (Feature Distance Multi Syllabic). In particular, we split the words into their constituent syllables, which we further split into their constituent segments onset, nucleus, and coda (see Table 1, rows 2 and 3), allowing for three phonemic slots for onset, two for vowels, and three for the coda per syllable. Syllables in monosyllabic words (see Table 1, row 1) are compared with one another by repositioning the phonemes of each syllable such that they result in the minimum distance between the phonemes of the two syllables. In order to calculate the distance between two phonemes, each phoneme is characterized by a feature vector (using the phoneme features provided by Stanford Phonology Archive (SPA) (2019) based on Kristoffersen (2000)), which allows computation of the difference between the value assigned to a feature for each filled phoneme slot. The Euclidean distance between the two phonemes is then computed as the square root of the sum of squared differences between the values of the features of the phonemes (c.f. Fig. 4). Next, the sum of the Euclidean distance between corresponding phonemes in each word is calculated. Furthermore, when two syllables have a different number of phonemes (e.g., *[bɛr]* and *[b{r{ə}}d]*), we take the phonemes which have not been included in the distance calculation at that point and calculate the mean of the distances between this phoneme's feature representation and all other phonemes' feature representations of the other word. For example, if we already compared /b/‐/r/, /ɛ/‐/ə/ and /r/‐/d/ of the words [bɛr] and [b{r{ə}}d], then the /b/ of [b{r{ə}}d] has not been included so far. We, therefore, calculate the Euclidean distances of /b/‐/b/, /b/‐/e/ and /b/‐/r/ and add the mean distance of them to the Euclidean distances we had already included (/b/‐/r/, /ɛ/‐/ə/ and /r‐d/). We constrain our alignment within onset, vowel, and coda segments. Thus, we always compare onsets with onsets, vowels with vowels, and codas with codas.^3^

**Table 1:** Example of how syllables were split into their constituent onset, vowels, and coda

Word/Syllable	Onset			Vowels		Coda		
skul	s	k	_	u	_	l	_	_
sofə	s	_	_	o	u	_	_	_
sofə	f	_	_	ə	_	_	_	_

*Note*. The words used here are [skul] and [soufə].

In considering multi‐syllabic words, additional computations were necessary. This is particularly important for words that contain the same or similar syllables, but do not have them in the same position in the word. For example, the words *football* and *ballgame* include the word *ball*. If we were to compare these two words syllable‐by‐syllable in the order in which they appeared in the words, we would compare *foot* with *ball* and *ball* with *game*, which results in the phonological distance being higher than it should be despite two syllables in the word being exactly the same. We, therefore, calculated the sum of the phonological similarity of all syllable permutations of word 1 and word 2 (i.e., [$FDMS_{foot-ball}$ + $FDMS_{ball-game}$], [$FDMS_{ball-ball}$ + $FDMS_{foot-game}$]), with the combination whose FDMS score is the lowest ([$FDMS_{ball-ball}$ + $FDMS_{foot-game}$) then serving as our final FDMS score.

If word 1 and word 2 do not have the same number of syllables, for example, *football* and *strawberry*, we calculate the phonological distance of all syllable permutations, that is, [$PT_{foot-straw}$ + $PT_{ball-ber}$], [$PT_{foot-straw}$ + $PT_{ball-ry}$], [$PT_{foot-ber}$ + $PT_{ball-straw}$], [$PT_{foot-ber}$ + $PT_{ball-ry}$], [$PT_{foot-ry}$ + $PT_{ball-straw}$], [$PT_{foot-ry}$ + $PT_{ball-ber}$]. The pairing with the lowest PT score is then the basis of further calculations. Thus, if [$PT_{foot-straw}$ + $PT_{ball-ber}$] has the lowest PT score of the six permutations noted above, we calculate the mean PT score of [$PT_{foot-ry}$] and [$PT_{ball-ry}$], which is then added to the PT score of [$PT_{foot-straw}$ + $PT_{ball-ber}$] for the final FDMS score.

## 2 Methods

### 2.1 Data

We used the Norwegian longitudinal vocabulary corpus (Words and Sentences) from wordbank (Frank, Braginsky, Yurovsky, & Marchman, 2017; Simonsen, Kristoffersen, Bleses, Wehberg, & Jørgensen, 2014). Relative to other languages that have been the focus of investigation on studies on the role of INT and EXT on vocabulary growth (e.g., English and French), Norwegian has a comparable phoneme inventory size (English: 41, French: 37, Norwegian: 40). Furthermore, despite there being few differences in the number of syllables per word (English: 1.32, French: 2.19, Norwegian: 1.6) and phonemes per syllable (English: 2.68, French: 2.47, Norwegian: 2.58) across the languages, there appear to be more phonemes per word in Norwegian (4.12), relative to English (3.4) and French (3.11) (Fenk‐Oczlon & Pilz, 2021). Thus, there may be slightly increased phonological complexity in words in Norwegian relative to other languages studied thus far. We downloaded the corpus using the R package wordbankr; all following steps were implemented in Python 3.8.2. The data frame contains data from 8753 observations of 4609 different children with regard to which of the 731 words (in the inventory) a child already produces at each individual observation. Since our focus was on longitudinal vocabulary development, we deleted all data from children with less than five observations, which resulted in us considering data from 326 children with 2155 observations in total, that is, completed CDIs (average observations: 6,61). The data contain 150 female and 176 male children. The age range of the children was 17–36 months (mean age: 26.27 months) over all observations, and the first observation took place between 17 and 29 months (average: 20.93 months). One hundred and thirty‐five children were first‐borns, 96 had one older sibling, 60 two older siblings, and 18 three or more older siblings. The mothers had different educational backgrounds, but were biased toward higher education (Graduate: 109, College: 165, Secondary: 49, Primary: 3; ordered by education level from high to low). Furthermore, given our focus on the phonological distance between words, we deleted all multi‐word expressions from the data (a whole list of deleted words can be found on OSF^4^), leaving us with data for 699 words for each child at each observation. We retrieved the IPA‐transcriptions of the words using the NLB Pronunciation Lexicon for Norwegian Bokmål (NLB Pronunciation Lexicon for Norwegian Bokmål, 2018). Those words missing from the lexicon were transcribed by hand by a Norwegian linguist (*n* = 26). For the phoneme features, we used the phoneme features provided by Stanford Phonology Archive (SPA) (2019), which is based on Kristoffersen (2000). For subsequent analyses, we used the frequency of words from the Norwegian Web as Corpus (Guevara, 2010). While frequency information was missing for some of the words included in the inventory, this concerned only 0.9% of the words (*n* = 6 words), which we do not anticipate influencing the models.

### 2.2 Creation of phonological networks

We created two types of networks, namely, a final network, which included all words of the CDI, and individual networks, which were the networks for each individual child at each observation. Prior research differs with regard to the thresholds according to which the connectedness of two words is assumed (Fourtassi, Bian, and Frank, 2020; Laing, 2024). We follow Laing (2024) and added edges between two nodes $n_{1}$, $n_{2}$ when the normalized $FDMS(n_{1},n_{2})\leq 0.25$. We used the package networkx from Python to create and analyze the networks.

### 2.3 Models

To determine whether phonological networks grow in an INT‐ or EXT‐like manner, we used the glmer function from the lme4 R library to fit a generalized linear mixed model with binomial as family argument and the link function logit. Our binary dependent variable “produced” captured whether a child was reported to produce a word in the observation under investigation (1) or not (0). Our main predictors were the INT and EXT values. We also added an interaction between our main predictor variables and age to examine the dynamics of growth scenarios best predicting vocabulary growth across development (Siew & Vitevitch, 2020a). In addition, we added multiple independent variables to our model which are known to influence word learning. As children learn shorter words earlier than longer words, and more frequent words earlier than less frequent words, and child's sex may influence the number of words known to children, we added sex, length, and frequency (based on NoWaC; Guevara, 2010) as predictors to our models. Furthermore, we controlled for the mother's education and the child's birth order in examining the extent to which the different growth scenarios captured vocabulary growth in individual children. In the predictor mother's education, we combined the levels “primary” and “secondary” to the level “school,” because only a few children had mothers who only completed primary education. The other two levels were “college” and “graduate” with the baseline college. For the predictor birth order, we combined the levels “fourth” and “sixth” to the new level “FourthPlus” as not many families had more than four children. Vocabulary growth research in the past has almost always only looked into nouns, since the first words of children are highly biased toward nouns (Fourtassi, Bian, and Frank, 2020; Hills, Maouene, Maouene, Sheya, and Smith, 2009; Siew and Vitevitch, 2020b). To control for this bias while including not only nouns in our models, we add a random intercepts effect of word category and a by‐category random slope for the effect of word frequency, because words from different categories differ in frequency. All models were fitted on data of children who were observed five or more times (overall 326 children) to capture vocabulary growth longitudinally. All quantitative predictor variables were centered. Full‐null model comparisons excluding critical predictor variables (INT and EXT) allowed us an estimate of the contribution of individual predictors to model fit. The syntax of our null model is reported in 1.

(1) $$\begin{array}{ccc} produced & ∼ & age+mom\_ed+birth\_order+length+frequency+sex\\ & & +\;(1+frequency||child)\\ & & +\;(1+frequency+age||category) \end{array}$$

To take the two different vocabulary growth scenarios into account, we added both the INT and EXT values as fixed effects including an interaction with age to Model 1. As INT and EXT were weakly correlated (0.28), we additionally fit the same model with either only INT or EXT as predictors (Model 3).

(2) $$\begin{array}{ccc} produced & ∼ & (INT+EXT)*age\\ & & +\;mom\_ed+birth\_order+length+frequency+sex\\ & & +\;(1+frequency||child)\\ & & +\;(1+frequency+age||category) \end{array}$$

(3) $$\begin{array}{ccc} produced & ∼ & INT\;\text{(or}\;EXT)*age)\\ & & +\;mom\_ed+birth\_order+length+frequency+sex\\ & & +\;(1+frequency||child)\\ & & +\;(1+frequency+age||category) \end{array}$$

After the models had been fitted,^5^ we compared the model fits against one another using Akaike weights (R library AICcmodavg), the likelihood ratio chi squared test (R library lmtest), and $R^{2}$‐values (R library performance). Note that this model differs from the preregistered model (see OSF), which failed to converge within a reasonable time despite using different optimizers (bobyqa, nloptwrap, Nelder_Mead, nlminbwrap, nmkbw, optimx.L‐BFGS‐B, nloptwrap.NLOPT_LN_NELDERMEAD, nloptwrap.NLOPT_LN_BOBYQA) and increasing the number of iterations. Considering the high complexity of the original models with multiple random effects, random slopes, and fixed effects, we simplified the preregistered model step‐by‐step by omitting less relevant random slopes and report the first model that converged within a reasonable time, that is, the model described above. The predictors “sex,” “birth_order,” and “mom_ed” were added following reviewers' comments.

## 3 Results

We will first describe the network which we created based on the Norwegian CDI from wordbank Frank, Braginsky, Yurovsky, & Marchman (2017), and report the results from the models afterward.

### 3.1 The network

**Fig. 5:**
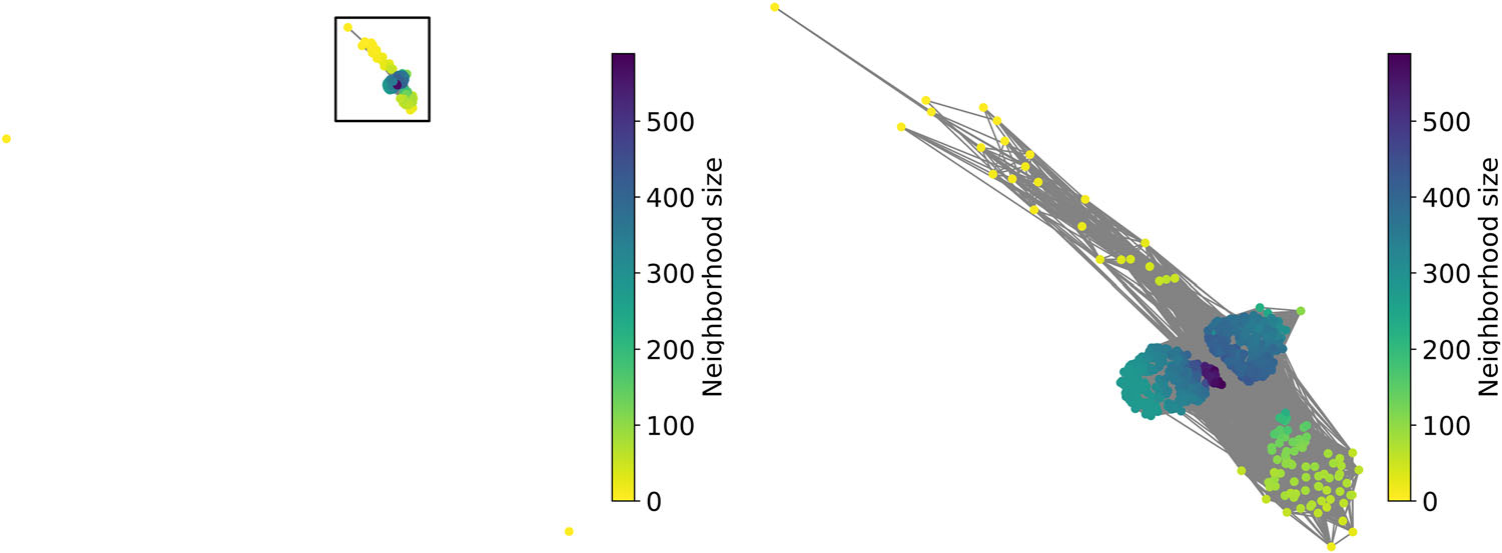
Final networks based on FDMS. On the left is the entire network, while the image on the right zooms into the rectangular area. Each dot represents a word; words which form a cluster are phonologically similar. Dots in lighter colors have fewer connections than darker dots. Average path length: 1,57; network density: 0.47; largest connected component: 697; number of hermits: 2; network diameter: 5.

Fig. 5 plots the final networks of Norwegian children using the words included in the Norwegian communicative vocabulary inventory (Simonsen, Kristoffersen, Bleses, Wehberg, & Jørgensen, 2014), capturing the phonological similarity of words as estimated by FDMS. The dots represent individual words, while the color of the dots indicates the connectedness of the words, with lighter colors indicating words with fewer neighbors and darker colors indicating words with many neighbors.

The network shows a dense structure with efficient connectivity. The presence of a significant largest connected component (697 words) and a few isolated hermits (2) indicates that while most words are well‐integrated into the lexicon, a few remain on the periphery. The average path length (1.57) and network diameter (5) suggest that words are generally easy to access, promoting quick retrieval.

### 3.2 The models

In what follows, we will first report goodness of fit for all fitted models. Afterward, we will show the results for the different predictors included in the models and discuss the differences between them.

#### 3.2.1 Goodness of fit

We compared the goodness of fit of all models, that is, the models including only INT, only EXT, or both INT and EXT as predictors, as well as the null model, using the Akaike Information Criterion (AIC) and Tjur's pseudo $R^{2}$‐values. The results are shown in Table 2 and demonstrate that all models increase the model fit compared to the null model. The $\chi^{2}$‐values of the comparison with the null models are also reported here. While the model including both INT and EXT as predictors fit the data better than the models with only one of the two predictors, that is, either INT or EXT, the model with only INT outperformed the model with only EXT (see Table 2). Furthermore, adding INT to the EXT model improved model fit more than adding EXT to the INT model. This is supported by the $\chi^{2}$‐values (Table 2), which suggested that the model including both INT and EXT as predictors was more similar to the model including just INT relative to the model including just EXT as a predictor. However, the overall differences between the models were small: While the null model explains 44.21% of the data, the model fit increases by 0.57–44.78% for the best model.

**Table 2:** AIC and $R^{2}$‐values of all models

Model	K	AICc	δ AICc	$\chi^{2}$ comp. w/ null model	$R^{2}$‐value	$\delta R^{2}$ to null model	$\chi^{2}$ comp. w/ INT+EXT model
INT+EXT	20	1,270,128	0	11,522	0.4478	0.0057	—
INT	18	1,270,342	214	11,304	0.4477	0.0056	218
EXT	18	1,280,858	10,730	788	0.4425	0.0004	10,734
Null	16	1,281,642	11,514	—	0.4421	—	11,522

*Note*. The model with the smallest AIC‐value, or with the largest $R^{2}$‐value, respectively, fit the data best. The models are sorted by AIC/ $R^{2}$‐values such that the model with the smallest value is in row 1 and the largest in the last row. δAICc shows the difference between the best fitting model and the model in the respective row. $\chi^{2}$‐values show the difference between all models and the null model or the INT+EXT model, respectively.

Overall, the model results, together with the comparisons to the null model, indicate that both INT and EXT influence word learning, and that a word's INT value was a better predictor of the likelihood of a word being learned next than the word's EXT value. Despite finding significant effects of INT and EXT, we note that the size of these effects was very small. In the following, we will take a closer look at the effects of individual predictors.

#### 3.2.2 Effect outcome

**Fig. 6:**
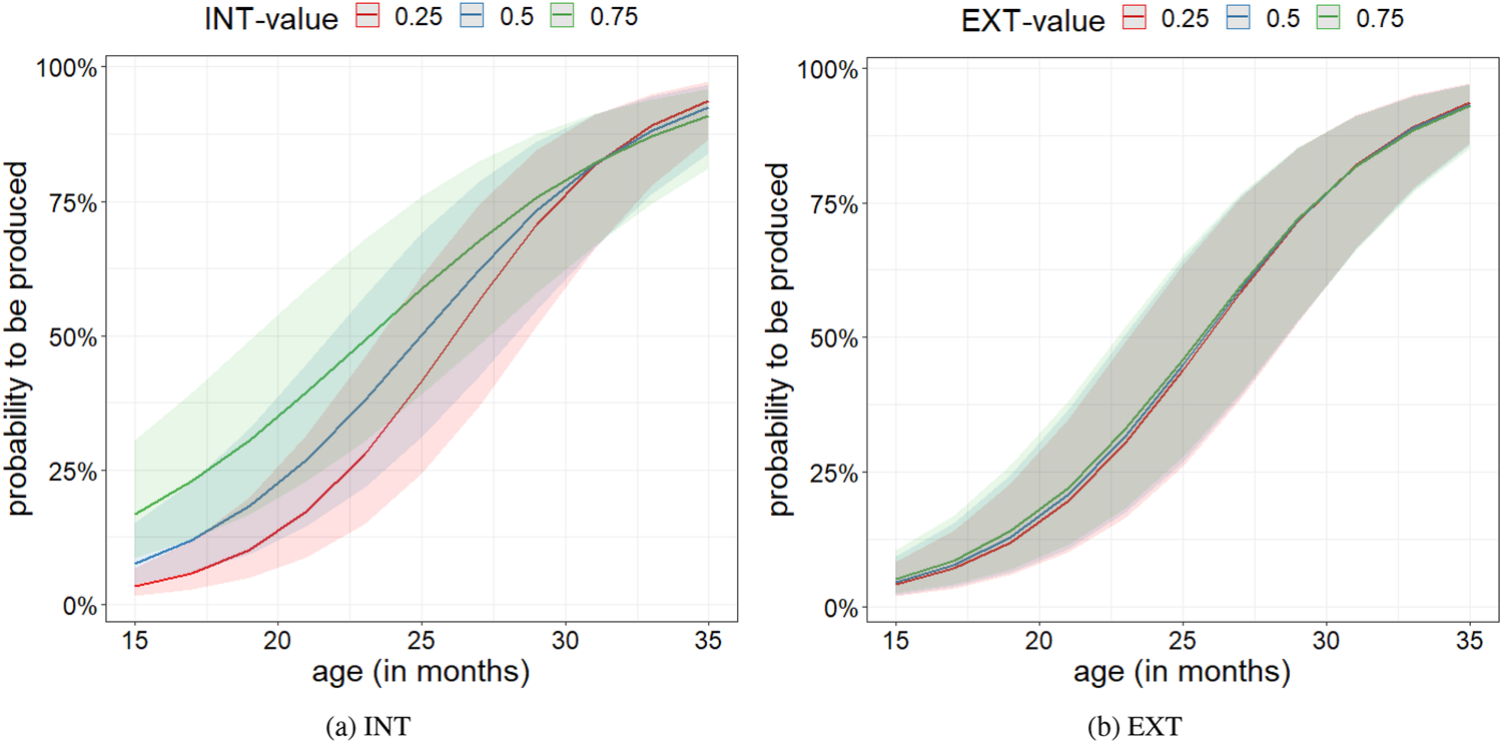
Effects of the predictors INT (a) and EXT (b) in the model which includes INT and EXT as predictors.

The estimates of the fixed effects are reported in Table 3. Note that we only present the results of the models including both INT and EXT as predictors here, since the results were similar across models. The effect outcomes in the INT‐only and EXT‐only models are presented in the Supplementary Materials. Differences in the effect outcomes across the models are highlighted. Regarding our critical predictors, both INT and EXT significantly predicted the probability of a word entering the lexicon. Words with higher INT values were more likely to be learned next compared to words with lower INT values, even after controlling for the effect of age. Fig. 6a plots the negative effect of the interaction between INT and age, with the gap between the three lines (indicating different INT groups) decreasing across development. Note that while Fig. 6a presents discrete INT value groups, the models included actual INT values for each word. In other words, at younger ages, a word's INT value better predicts the likelihood of that word entering the lexicon than at later ages, where the effect seems to reverse (Fig. 6a).

**Table 3:** Fixed effects of the model predictors of the INT+EXT models. In comparison to the baseline “College.” In comparison to the baseline “Female.” In comparison to the baseline “1st‐born”

Variable	INT+EXT Est	INT+EXT SE	*p*‐value
INT	3.59	0.03	<.001
EXT	0.50	0.03	<.001
Age	7.57	0.16	<.001
Mother's Ed. Grad. 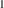	0.23	0.15	.13
Mother's Ed. School 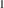	0.21	0.17	.23
Sex (male) 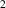	−0.46	0.14	<.01
2nd‐born 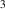	−0.22	0.15	.15
3rd‐born 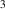	−0.24	0.19	.20
4th/6th‐born 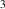	−0.37	0.23	.11
Length	−1.44	0.03	<.001
Frequency	4.61	0.37	<.001
INT:Age	−4.41	0.05	<.001
EXT:Age	−0.66	0.06	<.001

*Note*. Frequency, INT, and EXT variables are log‐transformed due to their skewed distribution. SE stands for standard error, Est for estimate.

Furthermore, this model suggests that the higher a word's EXT value was, the higher was the probability of this word being learned, although this effect was much smaller than the effect found for INT. As with INT, the influence of EXT decreased with age, although this effect was smaller than the interaction between INT and age (compare Table 3 and Fig. 6b).

**Fig. 7:**
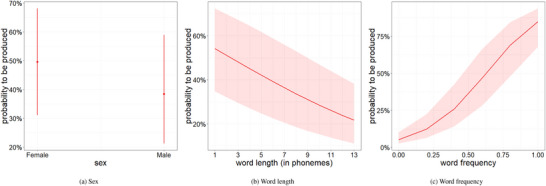
Effects of sex, word length, and word frequency (normalized) on the probability of a word being learned based on the INT&‐EXT model.

Finally, as expected and can be seen from Table 3 and Fig. 7, shorter and more frequent words were more likely to be learned next than longer and less frequent words. Girls also had a higher probability of a word being learned than boys (compare Fig. 7), echoing previous sex‐based differences in early word learning (Huttenlocher, Haight, Bryk, Seltzer, & Lyons, 1991; Rowe & Leech, 2017). The mother's educational attainment and the number of siblings did not have an effect on the word production probability.

## 4 Discussion

The current study set out to examine the extent to which the phonological connectivity between the words a child already knows (INT vocabulary growth scenario) and/or words in her environment (EXT vocabulary growth scenario) shapes the pattern of early vocabulary growth. We also examined the dynamics of these growth scenarios across development. In the following, we will first discuss our results and then compare our results with those of other studies to provide a more comprehensive picture of early vocabulary development.

Our results find strong support for INT‐like vocabulary growth: The model including only INT as a predictor outperformed the model including only EXT as a predictor, and adding INT to the EXT model improved model fit more than adding EXT to the INT model. We interpret these findings as suggesting that the early vocabulary grows in keeping with a rich‐get‐richer growth mechanism: Children build upon what they already know such that they are more likely to learn words that sound similar to already‐learned words, which are well‐connected to other similar‐sounding words in the lexicon. This pattern of results is typically explained with recourse to leveraging accounts (Barabási & Albert, 1999; Mani & Ackermann, 2018), according to which children leverage their current knowledge in acquiring novel information. Thus, greater familiarity with phonological patterns that repeat across many different words in their lexicon may lead to children attending more to such patterns in their input (Altvater‐Mackensen and Mani, 2013) and learning these words earlier (Newman, Samuelson, & Gupta, 2008). This paints a picture of the early vocabulary as a collection of clusters of words, with larger clusters attracting words overlapping in certain dimensions with members of the cluster to the lexicon. At the same time, our findings highlight the remarkable efficiency of early language development with each word learned acting as a stepping stone for subsequent learning, opening up pathways for a veritable explosion of language learning.

Nevertheless, our results did find some support for EXT‐like vocabulary growth, although INT‐like growth outperformed all EXT models. This is in keeping with an earlier study finding evidence for both growth patterns in vocabulary development for children between 3 and 9 years (Siew and Vitevitch, 2020b), which we extend to even younger children. Our findings speak to a multi‐mechanistic approach to language development, with clusters of words in the child's environment likely being either prioritized (by the caregiver) or attended to more (by the child) in the input, leading to words overlapping with such clusters being learned earlier. A role for EXT in semantic networks is typically explained by suggesting that words that overlap semantically often co‐occur together leading to clusters containing these words being thematically spotlighted in certain contexts and children learning words related to spotlighted clusters easily. The thematic spotlight further boosts learning by providing a context in which such words are heard, such as food‐related words being heard at mealtimes or in the kitchen (Roy, Frank, DeCamp, Miller, & Roy, 2015). A similar explanation may not hold for phonological networks—phonologically related words are not similarly likely to co‐occur together to the extent that semantically related words do—potentially reducing the role of EXT‐like growth patterns in phonological networks. However, an advantage of learning phonologically similar words lies in articulation. As we considered word production data in our study, a word is considered learned once a child produces it. Thus, a child must not only learn the word's meaning but also be able to produce specific sound combinations. By the ages under investigation, toddlers are still developing their phonological skills, particularly with complex sounds. While there is variation among children, typical speech sound development progresses significantly in this age range (Prather, Hedrick, & Kern, 1975). Similarity in sound can, therefore, be of leveraging nature—once a child can produce a certain word, it becomes easier to produce other words that sound similar. We note also that models including only EXT as a predictor revealed a larger positive association between increase in EXT values and the likelihood of words being learned than models including both INT and EXT as predictors. This is likely due to shared variance between INT and EXT predicting the likelihood of a word being learned, leading to a smaller role for EXT only variance on model fit in models including INT and EXT.

Importantly, both INT and EXT effects were most pronounced early in development and, while still influential, become less pronounced over time within our observed age range (15–35 months). This effect was more strongly pronounced for INT, where the effect also reverses later in development, such that higher INT values reduce the likelihood of a word being learned. This replicates the dynamics of growth principles found in other studies, where higher INT values better predict learning early in development, while lower INT values predict learning later in development (Luef, 2022; Siew & Vitevitch, 2020a; Siew and Vitevitch, 2020b). Siew & Vitevitch explain this pattern by suggesting that phonological similarity may aid word learning in the beginning due to familiar patterns repeating and leveraging learning. However, as children learn more words, the density of the phonological space may become an impediment, increasing processing costs for words with high INT values. Interestingly, Siew and Vitevitch (2020b) and Luef (2022) report a similar pattern in older participants with larger vocabularies compared to our investigated age group, that is, in 3‐ to 9‐year‐old children (Siew and Vitevitch, 2020b) and adult second language learners (Luef (2022)). This may seem counterintuitive, given that the oldest age‐group examined in the current study was the same age as the youngest age‐group tested in Siew and Vitevitch (2020b). Note, however, that INT values can only be calculated with regard to words included in the vocabulary questionnaires, and the oldest age included in the current study was the oldest age at which the questionnaire could be administered. Thus, children were likely to be at ceiling toward the end of the questionnaire, knowing most if not all the words in the questionnaire, thereby leading to a diminished INT effect. The same was true of the dataset used by Siew and Vitevitch (2020b), which only included words with AoA less than 10 years of age. This calls for caution in terms of attributing the changing pattern of results to changes in the algorithms governing learning across development.

Another interpretation for the reduction in the influence of INT growth scenarios on vocabulary development over time might be that children use phonological similarity to leverage learning early in lexical development. The focus on phonological similarity early on may help children detect frequent patterns in the input, refine their phonological representations of early words, and organize words along phonological dimensions with greater ease. Later on, with access to other kinds of information about words, for example, semantic similarity or category knowledge, children may enlist these other potentially more reliable cues to the service of learning. Were this to be true, the impact of phonological similarity on vocabulary development could be inversely related to the impact of semantic similarity on development at later ages. It could, therefore, be insightful to compare the impact of phonological and semantic similarity on word learning over time. Indeed, work by Stella, Beckage, and Brede (2017); Stella et al. (2024) suggests that combining multiple levels, capturing the semantic, phonological, and syntactic features of words, into a multi‐layer network better predicts learning relative to a single feature network. Alternatively, from a more generic standpoint, it may also be that older children rely less on leveraging their knowledge in later learning, relative to early language learners. Thus, children may begin learning words by building on the few words they already know. The more words children know, the more proficient they become and the less they rely on the words they know or the connectivity of words in their environment to build their vocabulary.

Our results agree—to a certain extent—with the results of prior research on this issue. Beckage and Colunga (2019) and Laing (2025) found that only INT was predictive of word learning in children, Luef (2022) found the same pattern of results in adult second language learners, while Fourtassi, Bian, and Frank (2020) argued that EXT but not INT predicts early vocabulary development. Siew and Vitevitch (2020b) report, similar to our findings, that both INT and EXT predict learning in older children, with INT having a stronger effect on the probability of a word being learned next relative to EXT. Our results support this latter interpretation using longitudinal data, that is, that both INT and EXT predict the growth of early networks, but that INT better predicts learning relative to EXT. In other words, while vocabulary growth is influenced both by connectivity of words already known to the child and words likely to be in the environment of the child (regardless of whether they are known to the child or not), there appears to be a stronger association between the likelihood of a word being learned and the connectivity of words known to the child that sound similar to this to‐be‐learned word.

The difference in the studies reported to‐date, including our study, could be explained by the data being examined across the studies. In particular, one potential reason why Fourtassi, Bian, and Frank (2020) did not find an effect of INT on vocabulary growth may be that they analyzed cross‐sectional data, that is, examined normative data of a generic child based on data of what words are typically known to children at different ages. In contrast, we examined longitudinal data of the words known to individual children at different points across development. Normative data may flatten out individual differences in the words known to different children at different time points across development. We extend the results of Laing (2025) to find a small but significant effect of EXT growth scenarios on vocabulary development. While Laing (2025) did not report a similar effect of EXT, the difference between our results may be explained either by the different languages under investigation, the fact that Laing (2025) examined actual productions of young children and we examined parental reports, or by the smaller number of children LaingLaing examined in her study (*n* = 9). Bringing together the literature to date, we find a consistent influence of INT on vocabulary growth, with a smaller effect of EXT, which appears contingent on the extent to which the data capture individual differences in vocabulary development and production. Indeed, INT and EXT‐like growth patterns need not be mutually exclusive. Thus, while the child may attend more to words in the input that are similar to words they already know, the environment may also be tailored to boost language learning by providing the child with input they can easily leverage and learn from (see also Amatuni & Bergelson, 2017, Fourtassi, Bian, and Frank, 2020, Siew and Vitevitch, 2020b for similar arguments).

We note that more nuanced insights into the phonological similarity of words could consider more detailed information about words. To avoid the use of normalization and thresholds, one could use weighted edges between nodes containing information about the strength of connections between words, that is, if words are extremely similar in phonology or just moderately similar, and the extent to which the strength of connections influences the pattern of vocabulary growth. The characteristics of the dataset may need more attention in planning future research on the factors influencing vocabulary growth. In particular, when investigating a theory of vocabulary growth across development, it may be critical to use data that reflects this period of development, that is, longitudinal data.

Finally, we note that, reassuringly, we replicate previous effects of the influence of the child's sex, word frequency, word length, and age of acquisition in our data. Thus, we found that female babies learn words earlier than male babies and that the probability of children learning a word increases with increasing age and higher frequency. Increases in word length, on the other hand, decrease the probability of children learning a word. Our replication of these effects speaks to the comparability of the data and methods employed in the current study to the literature thus far.

## 5 Summary

In the current study, we examined the factors influencing vocabulary growth, with regard to the likelihood of children learning specific words at different points across development. In particular, we examined the extent to which the likelihood of a child learning a word was influenced by the connectivity of similar words already known to the child relative to similar words in the environment of the child. Our results suggested that the early vocabulary grows predominantly in a rich‐get‐richer manner (INT), where word learning is predicted by the connectivity of words already known to the child. However, word learning is also, albeit to a lesser extent, influenced by the connectivity of words in the child's linguistic environment (EXT). Indeed, we found that the influence of the different growth scenarios varies dynamically across development, speaking to the possibility of multiple growth algorithms predicting vocabulary growth in early childhood.

With regard to future research, we see a clear need for larger samples of longitudinal data across languages, especially since we suspect that the differences in the results reported thus far may lie in the characteristics of the datasets being examined. In particular, we suggest that future research should examine this issue using longitudinal data from different languages. Indeed, we note that Laing (2025) reports differences between French and English, while the smaller differences between our results and Laing (2024) may be related either to differences in the sample size or the languages under investigation. At the same time, given the focus on semantic overlap in the development of theories of vocabulary growth, we see real promise in examining the extent to which similar patterns of vocabulary growth are found when examining the semantic connectivity of words already known to the child and in the child's environment. Ongoing research in our lab is currently examining this issue.[Supplementary-material cogs70109-supl-0001]

## Open Research Badges

This article has earned Open Data and Open Materials badges. Data and materials are available at http://wordbank.stanford.edu/data and https://osf.io/kd95f/.

## Supporting information

Table S1: Example of how syllables were split into their constituent onset, vowels, and coda which is used in the FD (and FDMS described below).
Figure S1: Example for the Euclidean distance of phoneme features based on FD.
Figure S2: Example for the Levenshtein distance (LD).
Table S2: Comparison of the properties of the different phonological distances.
Figure S3: Correlation between the three phonological distance measures.
Figure S4: Cut‐out of a phonological network based on the LD and a connection of words if their phonological distance is smaller or equal 2.
Figure S5: Final networks of three different phonological distances.
Table S3: Comparison of network metrics for LD, FDL, and FDMS networks.
Table S4: AIC and $R^{2}$ ‐values of all models.
Table S5: $\chi^{2}$ values of the different model comparisons.
Table S6: Fixed effects of the model predictors of the INT, EXT, and INT+EXT models based on the FDMS networks.
Table S7: Fixed effects of the model predictors of the INT, EXT, and INT+EXT models based on the FDL networks.
Table S8: Fixed effects of the model predictors of the INT, EXT, and INT+EXT models based on the LD networks.
Figure S6: Interaction of the predictors INT and EXT with age in INT‐ or EXT‐models.
Figure S7: Interaction of the predictors INT and EXT with age in INT+EXT models.
Figure S8: Impact of INT and EXT for each individual child (represented by a dot).
Figure S9: Number of words produced per child at first observation in the CDI.

## Notes

1 Other growth scenarios have also been discussed in past research, but only for semantic network growth, not for phonological networks.

2 Visualizations in Figs. 1, 2, 3 are inspired by Hills, Maouene, Maouene, Sheya, and Smith (2009).

3 Norwegian words are stressed on the first syllable, and stressed parts of words are included in word production more than unstressed syllables (Archibald, 1995). As the comparison of all possible syllable combinations between the word pairs does not keep the syllable order, we decided to not take this stress pattern into account.

4 The code can be found on the OSF (https://osf.io/kd95f/).

5 We fitted the same model based on different INT/EXT values computed using three different phonological distance measures. Find information about the distances, network structures, and model results in the Supplementary Materials.
