# Supplementary material for: The Development of Early Phonological Networks: An Analysis of Individual Longitudinal Vocabulary Growth
Source: Cogn Sci. 2025 Sep 1;49(9):e70109. doi: 10.1111/cogs.70109 (PMC12402682; doi:10.1111/cogs.70109)
Supplement: Supplementary file 1 — Table S1: Example of how syllables were split into their constituent onset, vowels, and coda which is used in the FD (and FDMS described below). Figure S1: Example for the Euclidean distance of phoneme features based on FD. Figure S2: Example for the Levenshtein distance (LD). Table S2: Comparison of the properties of the different phonological distances. Figure S3: Correlation between the three phonological distance measures. Figure S4: Cut‐out of a phonological network based on the LD and a connection of words if their phonological distance is smaller or equal 2. Figure S5: Final networks of three different phonological distances. Table S3: Comparison of network metrics for LD, FDL, and FDMS networks. Table S4: AIC and R2 ‐values of all models. Table S5: χ2 values of the different model comparisons. Table S6: Fixed effects of the model predictors of the INT, EXT, and INT+EXT models based on the FDMS networks. Table S7: Fixed effects of the model predictors of the INT, EXT, and INT+EXT models based on the FDL networks. Table S8: Fixed effects of the model predictors of the INT, EXT, and INT+EXT models based on the LD networks. Figure S6: Interaction of the predictors INT and EXT with age in INT‐ or EXT‐models. Figure S7: Interaction of the predictors INT and EXT with age in INT+EXT models. Figure S8: Impact of INT and EXT for each individual child (represented by a dot). Figure S9: Number of words produced per child at first observation in the CDI. [file COGS-49-e70109-s001.pdf]

Supplementary Materials: The development of early phonological networks: An analysis of individual longitudinal vocabulary growth

S-I. Phonological distance measures

As mentioned in the main manuscript, we examined three different distance measures of phonological similarity. This included the measure reported in the main manuscript (FDMS), which is an extended version of phoneme feature distance proposed by Monaghan et al. (2010), a second measure proposed by Laing (2024) (FDL, Feature Distance Laing), which also builds on Monaghan et al. (2010), and the Levenshtein distance. Past research on the development of early phonological networks has used different measures of phonological similarity, which could be one reason for the different results they report. To shed further light on this issue, we investigated whether there are differences in our findings based on the measure of phonological distance employed across different analyses. In the following, we first describe the different measures.

We will first describe the feature distance by Monaghan et al. (2010, Phonological Feature Distance, henceforth FD) and build on it with the subsequent approaches FDL and FDMS. Importantly, Monaghan et al. considered only mono-syllabic words. Words are transcribed according to IPA regulations. Each word is then split into its onset, vowels and coda, allowing for three phonemic slots for the onset, two for the vowels and three for the coda (see Table S1, row 1). Words are then compared with one another by repositioning the phonemes of the words such that they result in the minimum distance between the phonemes of the words. In order to calculate the distance between two phonemes, each phoneme is characterized by a feature vector (based on Harm & Seidenberg 1999), which allows computation of the difference between the value assigned to a feature for each filled phoneme slot. The Euclidean distance between the two phonemes is then computed as the square root of the sum of squared differences between the values of the features of the phonemes (cf. Figure S1). If words differ in phoneme length, e.g., [bivər] and [bɛr], additional phonemes are compared to empty phonemes, i.e., phonemes where all features are -1. Next, the sum of the Euclidean distance between corresponding phonemes in each word is calculated.

The phonological similarity measures proposed by (Laing, 2024, FDL) has strong similarities to FDMS (reported in the main manuscript). Both measures split the words into their syllables. Then, with some similarity to FDMS and FD, the vowels within the syllables of one word are mapped onto the vowels within the syllables of the other word, and the Euclidean distance between constituent phonemes is calculated (cf. Figure S1). However, there are three differences

Word/Syllable	Onset			Vowels		Coda		
skul	s	k	_	u	_	l	_	_
so(underlined)fə	s	_	_	o	u	_	_	_
sofə(underlined)	f	_	_	ə	_	_	_	_

Table S1: Example of how syllables were split into their constituent onset, vowels and coda which is used in the FD (and FDMS described below). The words used here are [skul] and [soufə].

$$
\begin{array}{cc|cc|cc}
\underline{\text{k}} & \textbf{k} & \underline{\text{ae}} & \textbf{u} & \underline{\text{t}} & \textbf{p} \\
\begin{pmatrix} f_1 \\ . \\ . \\ . \\ f_{11} \end{pmatrix} & \begin{pmatrix} f_1 \\ . \\ . \\ . \\ f_{11} \end{pmatrix} & \begin{pmatrix} f_1 \\ . \\ . \\ . \\ f_{11} \end{pmatrix} & \begin{pmatrix} f_1 \\ . \\ . \\ . \\ f_{11} \end{pmatrix} & \begin{pmatrix} f_1 \\ . \\ . \\ . \\ f_{11} \end{pmatrix} & \begin{pmatrix} f_1 \\ . \\ . \\ . \\ f_{11} \end{pmatrix} \\
\multicolumn{2}{c|}{FD_{k,k} = \sqrt{\sum_i (f_{i_k} - f_{i_k})^2}} & \multicolumn{2}{c|}{FD_{ae,u} = \sqrt{\sum_i (f_{i_{ae}} - f_{i_u})^2}} & \multicolumn{2}{c}{FD_{t,p} = \sqrt{\sum_i (f_{i_t} - f_{i_p})^2}}
\end{array}
$$

$$FD_{cat,cup} = \frac{FD_{k,k} + FD_{ae,u} + FD_{t,p}}{max(len(cat), len(cup))}$$

Figure S1: Example for the Euclidean distance of phoneme features based on FD. The phonemes of the words cat and **cup** next to each other with their 11 phonological features $f_1, ...f_{11}, f_i \in \{-1, 0, 1\}$ (based on Harm & Seidenberg 1999 The Euclidean distance between each phoneme pair is calculated.

$$
\begin{array}{ccccc}
\text{b} & \text{i} & \text{v} & \text{ə} & \text{r} \\
 & \downarrow & \downarrow & \downarrow & \downarrow \\
\text{b} & \text{ɛ} & \text{r} & &
\end{array}
\quad \Rightarrow \text{LD(bivər, bɛr)} = 4
$$

Figure S2: Example for the Levenshtein distance (LD). The word [bivər] needs four changes (visualised by arrows) to be converted into the word [bɛr]; the b does not have to be changed, i and v have to be substituted by b and ɛ, and ə and r must be deleted. All changes add up to 4. Thus, LD(cat, biver) = 4.

between FDL and FDMS. First, Laing (2024) does not include vowel features into her analysis. Second, FDL does not examine all syllable permutations but compares syllables in the order in which they appear in the word. Similar or even identical syllables are, therefore, not compared with each other unless they are in the same position within the words. Third, FDL does not realign the phonemes within the syllable's constituent onset, nucleus and coda segments. This leads to differences in cases where two words or two syllables to be compared have a different number of phonemes or have identical phonemes in different positions with the constituent onset, nucleus or coda segments (e.g., position of k in skuːl and in kaet). In particular, FDL adds 'empty phonemes' to the shorter word, i.e., phonemes where all features are zero, until the words have the same phonemic length. The last phonemes of the longer words/syllables are then compared to empty phonemes in the shorter words/syllables. Therefore, the FDL between two words is the sum of the Euclidean distance between the phonemes in each word (including empty phonemes, but excluding vowels).

A final index of phonological distance between two words is the Levenshtein distance (LD). As Table S2 suggests, there are few commonalities between the LD and the other methods reviewed above. However, potentially owing to its' ease of implementation and computation, the LD has been used frequently in prior research (Heeringa, 2004; Schepens et al., 2012; Fourtassi et al., 2020). Words are first transcribed according to IPA regulations. The LD then estimates the total number of steps required to convert a word, phoneme by phoneme, into the other word. Here, the removal, addition, or substitution of a phoneme (see Figure S2) constitutes a step. The LD does not consider phoneme features, and may, therefore, not be able to capture the

Property	LD	FDL	FDMS
Phoneme features	×	✓	✓
Vowel inclusion	✓	×	✓
Onset-Vowel-Coda alignment	×	✓	✓
Syllable permutation	×	×	✓
Onset-Vowel-Coda shifting	×	×	✓

Table S2: Comparison of the properties of the different phonological distances.

phonological distance between word pairs as accurately as the other two measures described above: For instance, the words *map*, *nap* and *cap* all differ by only one phoneme and thus have the exact same LD. However, due to the degree of feature overlap between their onset consonants, the words *map* and *nap* sound more similar to one another than the words *map* and *cap* or *nap* and *cap*, respectively (/m/ and /n/ share voicing, nasality, and manner of articulation unlike /n/-/k/ and /m/-/k/). Furthermore, the LD does not align the constituent onset, nucleus and coda segments of the two words and does not use syllable permutation or the realignment of phonemes within the syllables' constituent segments.

In what follows next, we compare the estimates of phonological distance provided by the three different measures (FDMS, FDL and LD) for all word pairs in the Norwegian vocabulary corpus (Words and Sentences, Simonsen et al. 2014). This allows us to examine the convergent validity of the estimates of phonological distance provided by the different measures.

S-I.1. Application of the phonological distance measures to our data

Our analysis of the convergent validity of the different measures of phonological distance compared the estimates provided by FDMS, FDL and LD. Their properties are compared in Table 2. Since the syllable structure of Norwegian consists of onset, nucleus and coda (Kristoffersen, 2000), as in English, and the IPA transcriptions provided the words' syllable boundaries, we were able to compute the phonological distances for Norwegian in a similar manner.

While all three measures were strongly correlated with one another (Pearson's correlation coefficient between two different measures of phonological distance of all possible word pairs of the 699 words; median correlation above 0.6), the correlation between LD and FDMS was the weakest of the three (median=0.6). LD and FDL were the most strongly correlated (median=0.8), while the median correlation between FDL and FDMS was slightly lower. We note, however, that the interquartile range was small for the FDL/FDMS correlation as compared to LD/FDL and especially for LD/FDMS; the same was the case for the whiskers (cf. Figure S3). This suggests that the variability between the phonological distances per measure is greater for LD/FDMS than for FDL/FDMS, and that, therefore, FDL and FDMS are more similar to one another than LD and FDMS.

In the main manuscript, we examine the influence of phonological distance between words as estimated by FDMS on the acquisition of similar-sounding words. In particular, we examined the extent to which the phonological distance of a to-be-learned word to words already in the child's lexicon or to words in the child's input influences the ease of acquisition of the word, as indexed

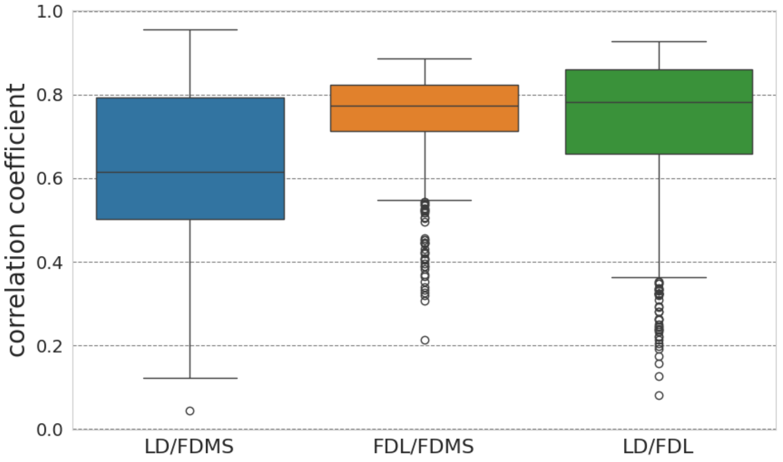

Figure S3: Correlation between the three phonological distance measures. The horizontal line in the boxplot shows the median. The whiskers show the range of typical data points within 1.5 times the Interquartile Range (IQR). They indicate the furthest data points within this range that are not considered outliers, which are plotted individually beyond the whiskers.

by the word's entry into the child's lexicon at the next observation. In this supplementary part, we do the same for the other two similarity measures, i.e., LD and FDL. Since such estimates depend on the phonological relations between all the words known to the child, i.e., the phonological networks in a child's lexicon, next, we provide a description of phonological networks and how they are typically inferred.

S-II. LD, FDL and FDMS networks

Prior research differs with regards to the thresholds according to which the connectedness of two words is assumed: Laing (2024) added edges between two nodes n_1, n_2 when the normalized $FDL(n_1, n_2) \leq 0.25$, while Fourtassi et al. (2020) added edges between nodes when the $LD(n_1, n_2) \leq 2$. In keeping with Fourtassi et al. and Laing, we will use a threshold of 2 to add edges between words in the LD networks, and a threshold of 0.25 in the FDL network. Consistent with Laing, we used the same threshold for the FDMS networks. We note that Laing normalized the distances between word pairs within each child by dividing by the largest distance of all word pairs the child had known at each observation point. In contrast, with the FDMS measure, we normalized the distances by dividing by the largest distance of all word pairs in the CDI. This allowed us to ensure that the phonological distance between word pairs does not change across development. This way of normalization differs, however, only in the construction of *individual* networks because the final networks do not depend on individual children.

As one example of a phonological network, Figure S4 displays a cut-out of a phonological network based on the LD and the connectedness of nodes based on the threshold of $dist(n_i, n_j) = 2$. Dots represent words and the lines between the words stand for phonological connections between these words. Despite claiming that LD is the least detailed measure of connectedness, we chose to use the LD network for this example, because it is less crowded than the networks

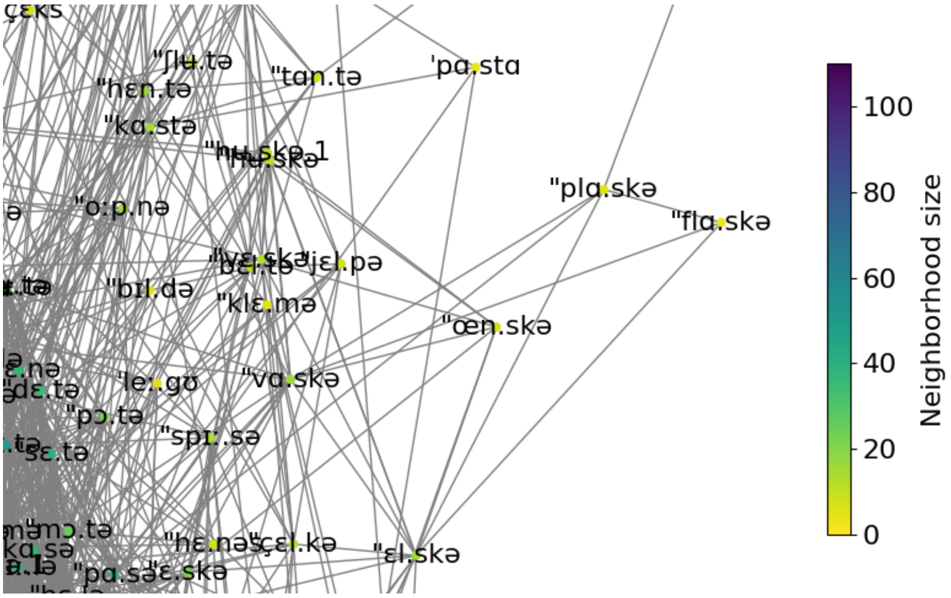

Figure S4: Cut-out of a phonological network based on the LD and a connection of words if their phonological distance is smaller or equal 2.

which use FDL or FDMS, where it would be more difficult to see the nodes and edges in detail.

Figure S5 plots the final networks of Norwegian children using the words included in the Norwegian communicative vocabulary inventory (Simonsen et al., 2014) as calculated by FDL, FDMS and LD. The dots represent individual words while the color of the dots indicate the connectedness of the words, with lighter colors indicating words with fewer neighbors and darker colors indicating words with many neighbors. As shown in the final networks in Figure S5 and Table S3, the three networks differ in their structure from each other with the FDL- and FDMS-network being more similar to each other than to the LD-network.

The LD network is the sparsest and most fragmented of all three networks, with the highest average path length and the largest number of isolated words. This indicates that fewer words are considered similar, leading to a more disjointed network structure. The large diameter suggests that the network is less efficient for spreading information or influence across all words.

The FDL network is denser and more connected than the LD network. The smaller number of hermits and a higher percentage of data following a power-law distribution indicate that certain words act as hubs, connecting many other words. The moderate average path length and diameter imply that the network is more efficient than the LD network but not as tightly connected as the FDMS network.

The FDMS network is the densest and most interconnected. It has the shortest average path length and diameter, suggesting highly efficient connectivity among words. The high adherence to a power-law distribution and minimal number of hermits indicate a few words serve as central hubs, integrating the vast majority of the lexicon into a single connected component. This network structure is ideal for rapid dissemination of information and reflects a robust vocabulary network where phonological similarity strongly binds words together.

The structure of the vocabulary networks varies significantly based on the similarity measures. The LD network is sparse and fragmented, reflecting stricter similarity criteria. The FDL

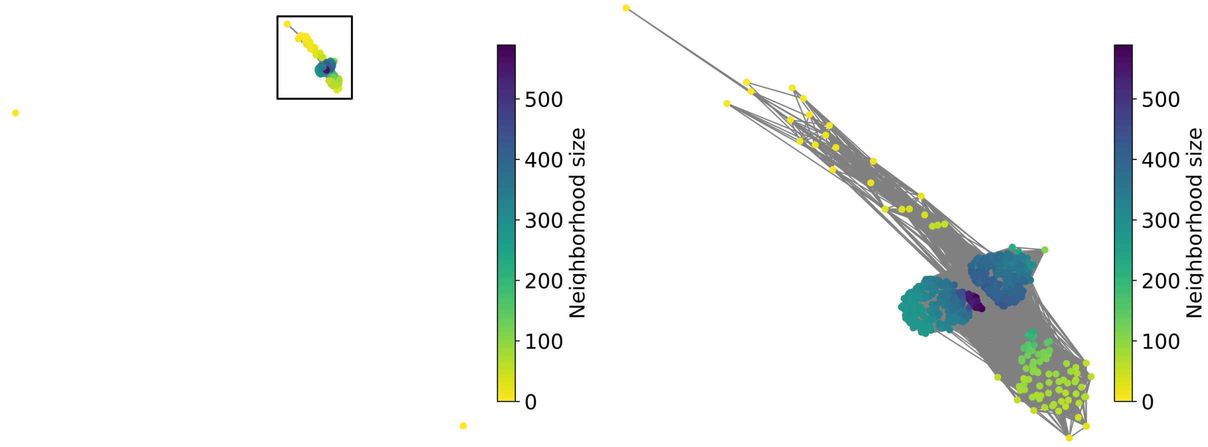

(a) FDMS

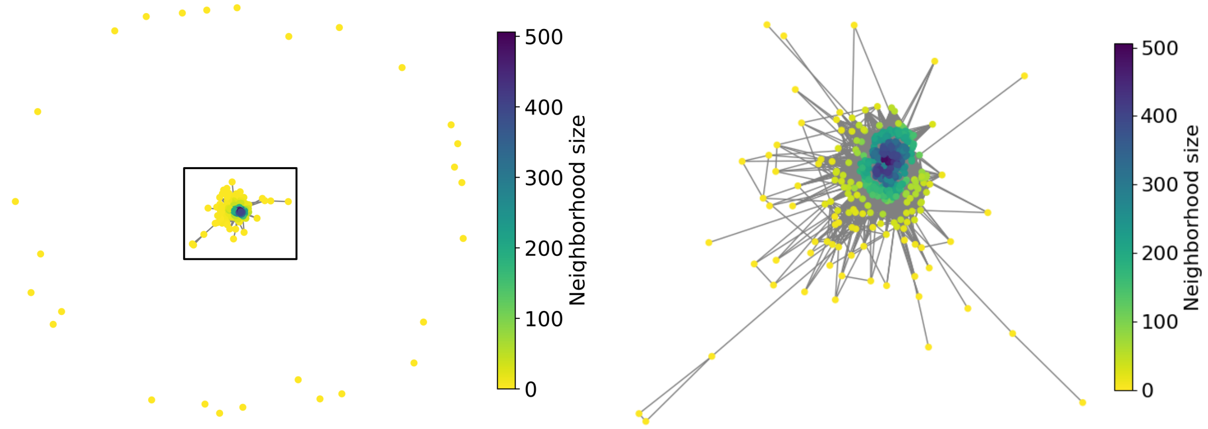

(b) FDL

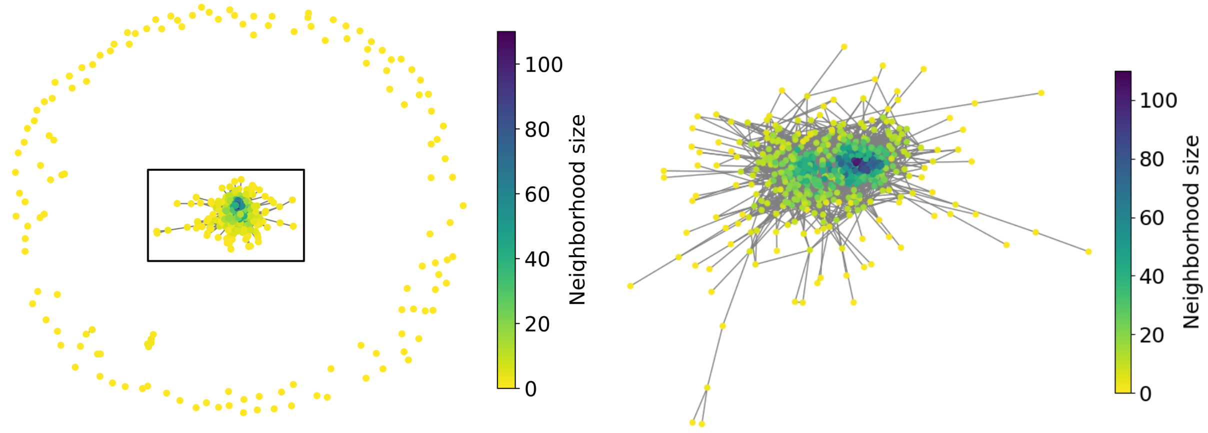

(c) LD

Figure S5: Final networks of three different phonological distances. On the left you can see the entire network while the image on the right zooms into the rectangular area. Each dot represents a word; words which form a cluster are phonologically similar. Dots in lighter colors have fewer connections than darker dots.

	LD Network	FDL Network	FDMS Network
Average Path Length	2.83	1.75	1.57
Network Density	0.0338	0.3449	0.4697
Power-law Distribution	8.2%	23%	52%
Largest Connected Component	556	671	697
Number of Hermits	132	28	2
Network Diameter	10	6	5
Number of connected components	137	29	3

Table S3: Comparison of network metrics for LD, FDL, and FDMS networks

network is moderately dense and well-connected, showing a balance between strict and relaxed similarity measures. The FDMS network is highly dense and almost fully connected, indicating very relaxed similarity criteria, resulting in a highly integrated vocabulary network. Normalizing the distances impacts the connectedness of the networks strongly: for instance, the largest distance value in FDMS was 140, while in FDL it was 61. Dividing other distances by 140 relative to 61 leads to more word pairs being under the threshold of 0.25 in the FDMS network, resulting in more connected words in the FDMS network compared to the FDL network.

In the context of vocabulary development, the FDMS network might reflect a more natural and fluid organization of words in a child's lexicon, where words are highly interconnected based on phonological similarity, facilitating easier word retrieval and learning. The LD network might represent a more constrained view, highlighting core phonological similarities but missing broader connections. However, the psychological reality of the different network properties, based on the extent to which words estimated to be more similar by FDMS relative to LD and FDL are actually perceived as similar remains to be ascertained.

S-III. LD, FDL and FDMS models

In this section, we compare models which are based on the three different distance measures. Please note that we did not use the same model structure as the model that we discuss in the associated paper but another model which has been changed during the revision process. This model has the following structure:

$$produced \sim (INT+EXT)(age+birthorder+motherseducation)+sex+length+frequency+(1+frequency||chi \tag{S1}$$

While model S1 does not have the same model structure, i.e. it includes an interaction of the factors mother's education and birth order with INT and EXT values, whereas the model in the associated paper only includes these effects without the interaction, it still serves the aim to detect differences that are due to the different distance measures.

We fitted model S1 using INT and EXT based on FDMS, LD and FDL networks. In what follows, we will first report goodness of fit for all fitted models. For reasons of comparability, we will present the results of all three measures. Afterwards, we will show the results for the

Model	K	AICc	δ AICc	χ^2 comparison with null model	R^2-value	δR^2 to null model
FDMS	32	1269708	0	11966	0.4480	0.0059
FDL	32	1269788	80	11886	0.4475	0.0054
INT FDMS	24	1269984	276	11674	0.4478	0.0057
INT FDL	24	1270539	832	11118	0.4471	0.0050
LD	32	1272548	2840	9126	0.4465	0.0044
INT LD	24	1273269	3561	8389	0.4462	0.0041
EXT LD	24	1279179	9472	2479	0.4433	0.0012
EXT FDL	24	1280010	10302	1648	0.4429	0.0008
EXT FDMS	24	1280842	11135	816	0.4425	0.0004
Null	16	1281642	11934	-	0.4421	-

Table S4: AIC and R^2-values of all models. The model with the smallest AIC-value, or with the largest R^2-value respectively, fits the data best. The models are sorted by AIC/R^2-values such that the model with the smallest value is in row 1 and the largest in the last row. $\delta AICc$ shows the difference between the best fitting model and the model in the respective row. χ^2-values show the difference between all models and the null model. Note that models which do not include INT or EXT in their name include both INT and EXT as predictors.

different predictors included in the models and discuss the differences between them.

S-III.1. Goodness of fit

We compared the goodness of fit of all models, i.e., for each of the three distance measures as well as the null model, using the Akaike Information Criterion (AIC) and Tjur's pseudo R^2-values. The results are shown in Table S4 and demonstrate that all models increase the model fit compared to the null model. The χ^2-values of the comparison with the null models are also reported here. While the models including both INT and EXT as predictors fit the data better than the models with only one of the two predictors, i.e., either INT or EXT, the models with only INT outperform the respective models with only EXT models in all cases (see Table S4). Furthermore, adding INT to the EXT model improved model fit more than adding EXT to the INT model. This is supported by the χ^2-values (Table S5), which suggest that the model including both INT and EXT as predictors is more similar to the model including just INT relative to the model including just EXT as a predictor. Regarding the different phonological distance measures, the AIC- and R^2-values find that the FDMS measure results in the best model fit, followed by the FDL model and the LD model as poorest performer. However, the overall differences between the models are very small: While the null model explains 44.21% of the data, the model fit increases by 0.59% to 44.80% for the best model, i.e., the FDMS model.

Overall, the model results, together with the comparisons to the null model, indicate that both INT and EXT influence word learning, and that a word's INT value is a better predictor of the likelihood of a word being learned next than the word's EXT value. Despite finding significant effects of INT and EXT, we note that size of these effects size was very small. In the following, we will take a closer look at the effects of individual predictors.

Model	Both	FDMS	FDL	LD
FDMS	-	-	80	2840
FDL	-	80	-	2760
INT FDMS	292	-	556	3285
INT FDL	767	556	-	2730
LD	-	2840	2760	-
INT LD	737	3285	2740	-
EXT LD	6648	1663	831	-
EXT FDL	10238	832	-	831
EXT FDMS	11151	-	832	1663

Table S5: χ^2 values of the different model comparisons. The first column presents model comparisons of the respective INT or EXT models with the model including both INT and EXT. For example, the INT FDMS model is compared to the (INT + EXT)-FDMS model. The second column presents model comparisons with the respective FDMS models. For example the INT-LD model is compared to the FDMS-INT model. The third and fourth columns similarly presents model comparisons of the respective models to the FDL and LD models.

S-III.2. Effect Outcome

Despite the fact that some models fitted the data better, there were almost no differences in the pattern of the results regarding individual predictors across the three phonological distance measures. The estimates of the fixed effects for all ten models are reported in Table S6 (for the FDMS models), Table S7 (for the FDL models) and Table S8 (for the LD models); the respective plots can be found below in Figures S6 and S7. As the results of the LD and FDL models were very similar to the results of the FDMS models, we refer the reader to the results section of the corresponding paper.

Variable	INT Est	INT SE	EXT Est	EXT SE	INT+EXT Est	INT+EXT SE
INT	3.45	0.04	-	-	3.37	0.04
EXT	-	-	0.85	0.04	0.59	0.04
Age	7.27	0.16	7.23	0.13	7.59	0.16
School[1]	*0.24*	*0.18*	*0.20*	*0.21*	*0.22*	*0.20*
Graduate[1]	*0.19*	*0.15*	*0.26*	*0.16*	*0.21*	*0.16*
Sex (male)[2]	-0.45 (**)	0.14	-0.56	0.16	-0.46 (**)	0.14
2nd-born[3]	-0.29 (*)	0.14 (*)	*-0.24*	*0.17*	-0.26 (·)	0.15 (·)
3rd-born[3]	-0.31 (·)	0.18 (·)	*-0.24*	*0.20*	-0.29 (·)	0.17 (·)
4th/6th-born[3]	-0.48 (*)	0.23 (*)	*-0.39*	*0.26*	-0.44 (·)	0.23 (·)
Length	-1.61	0.02	-1.62	0.03	-1.44	0.03
Frequency	4.59	0.27	4.54	0.28	4.61	0.30
INT:Age	-4.59	0.05	-	-	-4.50	0.05
EXT:Age	-	-	-0.75	0.06	-0.63	0.06
INT:School	-0.21	0.03	-	-	-0.24	0.03
INT:Graduate	0.17	0.02	-	-	0.20	0.03
EXT:School	-	-	0.07 (*)	0.04 (*)	0.11 (**)	0.04 (**)
EXT:Graduate	-	-	*-0.04*	*0.03*	-0.11	0.03
INT:2nd-born	0.32	0.03	-	-	0.36	0.03
INT:3rd-born	0.39	0.03	-	-	0.42	0.03
INT:4th/6th-born	0.58	0.05	-	-	0.63	0.05
EXT:2nd-born	-	-	-0.10	0.03	-0.15	0.03
EXT:3rd-born	-	-	-0.08 (*)	0.03 (*)	-0.14	0.03
EXT:4th/6th-born	-	-	-0.15 (**)	0.05 (**)	-0.24	0.06

Table S6: Fixed effects of the model predictors of the INT, EXT, and INT+EXT models based on the FDMS networks. All values are significant, except for those which are printed in italics (p>0.1). Significance levels: '·' p<0.1, '*' p<0.05, '**' p<0.01, all other values p<0.001. Frequency, INT- and EXT-variables are log-transformed due to their skewed distribution. [1]In comparison to the baseline 'College'. [2]In comparison to the baseline 'Female'. [3]In comparison to the baseline '1st-born'. SE stands for standard error, Est for estimate.

Variable	INT Est	INT SE	EXT Est	EXT SE	INT+EXT Est	INT+EXT SE
INT	1.97	0.02	-	-	1.94	0.03
EXT	-	-	0.74	0.03	0.23	0.03
Age	7.34	0.16	7.03	0.13	7.25	0.15
School[1]	*0.18*	*0.17*	*0.22*	*0.20*	*0.19*	*0.19*
Graduate[1]	*0.21*	*0.16*	*0.25*	*0.17*	*0.21*	*0.15*
Sex (male)[2]	-0.47	0.14	-0.55	0.15	-0.47	0.13
2nd-born[3]	-0.28 (·)	0.16 (·)	*-0.27*	*0.17*	-0.23 (·)	0.16 (·)
3rd-born[3]	-0.29 (·)	0.17 (·)	*-0.26*	*0.19*	-0.30 (·)	0.18 (·)
4th/6th-born[3]	-0.46 (*)	0.20 (*)	-0.43 (·)	0.23 (·)	-0.4 (*)	0.23 (*)
Length	-0.99	0.03	-1.25	0.03	-0.49	0.03
Frequency	4.61	0.25	4.51	0.26	4.59	0.30
INT:Age	-2.33	0.04	-	-	-2.43	0.04
EXT:Age	-	-	-0.43	0.04	0.41	0.04
INT:School	0.05 (*)	0.03 (*)	-	-	*0.04*	*0.03*
INT:Graduate	0.07	0.02	-	-	0.09	0.02
EXT:School	-	-	0.09	0.03	*0.03*	*0.03*
EXT:Graduate	-	-	*-0.02*	*0.02*	-0.06 (**)	0.02 (**)
INT:2nd-born	0.12	0.02	-	-	0.17	0.02
INT:3rd-born	0.19	0.02	-	-	0.23	0.03
INT:4th/6th-born	0.32	0.04	-	-	0.32	0.04
EXT:2nd-born	-	-	-0.05 (·)	0.02 (·)	-0.13	0.02
EXT:3rd-born	-	-	*-0.03*	*0.02*	-0.12	0.03
EXT:4th/6th-born	-	-	*0.06*	*0.04*	*-0.04*	*0.04*

Table S7: Fixed effects of the model predictors of the INT, EXT, and INT+EXT models based on the FDL networks. All values are significant, except for those which are printed in italic (p>0.1). Significance levels: '·' p<0.1, '*' p<0.05, '**' p<0.01, all other values p<0.001. Frequency, INT- and EXT-variables are log-transformed due to their skewed distribution. [1]In comparison to the baseline 'College'. [2]In comparison to the baseline 'Female'. [3]In comparison to the baseline '1st-born'. SE stands for standard error, Est for estimate.

Variable	INT Est	INT SE	EXT Est	EXT SE	INT+EXT Est	INT+EXT SE
INT	1.80	0.03	-	-	2.16	0.04
EXT	-	-	0.84	0.03	-0.48	0.04
Age	7.04	0.15	6.93	0.13	6.83	0.15
School[1]	*0.22*	*0.20*	*0.23*	*0.20*	*0.22*	*0.20*
Graduate[1]	*0.24*	*0.17*	*0.25*	*0.15*	*0.24*	*0.16*
Sex (male)[2]	-0.453	0.15	-0.55	0.16	-0.52	0.14
2nd-born[3]	*-0.26*	*0.17*	-0.28 (·)	0.16 (·)	-0.26 (·)	0.15 (·)
3rd-born[3]	*-0.25*	*0.17*	*-0.26*	*0.18*	*-0.35*	*0.17*
4th/6th-born[3]	*-0.39*	*0.26*	-0.43 (·)	0.22 (·)	-0.39 (·)	0.23 (·)
Length	-0.62	0.03	-0.83	0.03	-0.61	0.03
Frequency	4.40	0.29	4.37	0.31	4.38	0.28
INT:Age	-1.74	0.04	-	-	-2.94	0.06
EXT:Age	-	-	-0.20	0.04	1.56	0.06
INT:School	*0.03*	*0.02*	-	-	*0.05*	*0.04*
INT:Graduate	0.05 (*)	0.02 (*)	-	-	0.12	0.03
EXT:School	-	-	0.10	0.02	0.11 (**)	0.04 (**)
EXT:Graduate	-	-	*<0.01*	*0.02*	-0.10	0.03
INT:2nd-born	*0.03*	*0.02*	-	-	0.18	0.03
INT:3rd-born	0.10	0.02	-	-	0.31	0.04
INT:4th/6th-born	0.22	0.04	-	-	0.47	0.06
EXT:2nd-born	-	-	-0.07	0.02	-0.19	0.03
EXT:3rd-born	-	-	-0.05	0.02	-0.27	0.04
EXT:4th/6th-born	-	-	*-0.02*	*0.04*	-0.33	0.06

Table S8: Fixed effects of the model predictors of the INT, EXT, and INT+EXT models based on the LD networks. All values are significant, except for those which are printed in italic (p>0.1). Significance levels: '·' p<0.1, '*' p<0.05, '**' p<0.01, all other values p<0.001. Frequency, INT and EXT variables are log-transformed due to their skewed distribution. [1]In comparison to the baseline 'College'. [2]In comparison to the baseline 'Female'. [3]In comparison to the baseline '1st-born'. SE stands for standard error, Est for estimate. Note that the INT+EXT model did not converge with max|grad| = 0.00209319 (tol = 0.002)

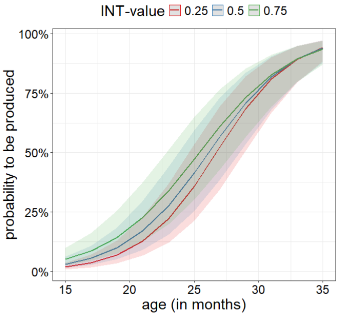

(a) FDL

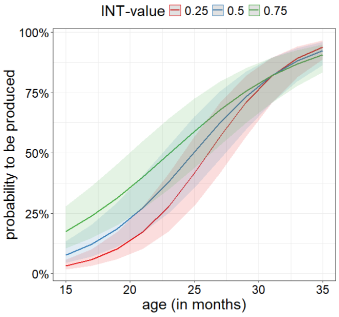

(b) FDMS

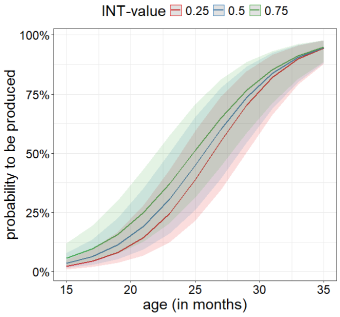

(c) LD

(i) Interaction of INT and age in INT-models

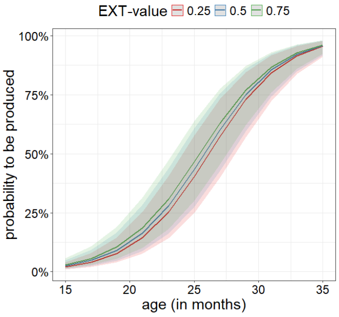

(a) FDL

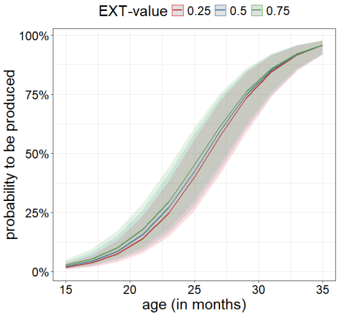

(b) FDMS

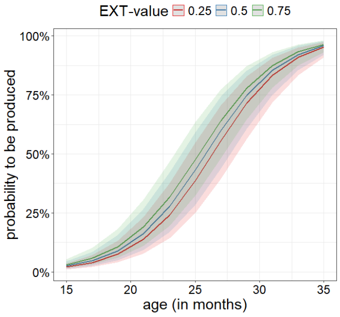

(c) LD

(ii) Interaction of EXT and age in EXT-models

Figure S6: Interaction of the predictors INT and EXT with age in INT- or EXT-models.

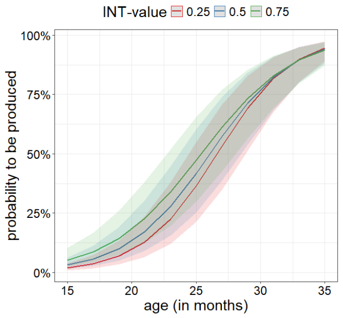

(a) FDL

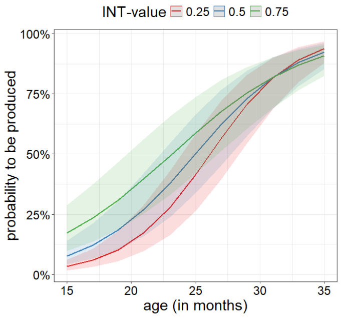

(b) FDMS

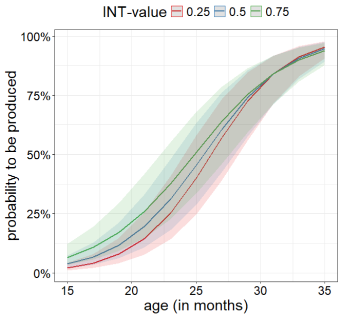

(c) LD

(iii) Interaction of INT and age in INT+EXT-models

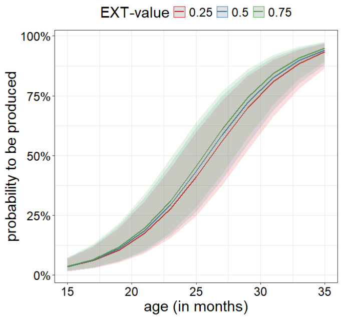

(a) FDL

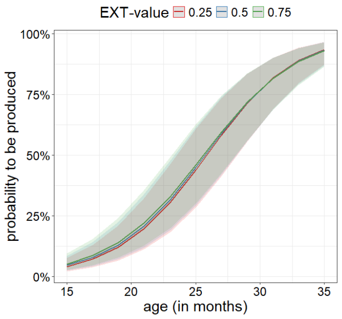

(b) FDMS

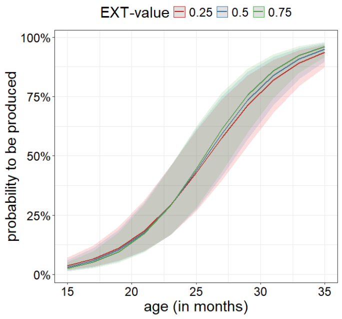

(c) LD

(iv) Interaction of EXT and age in INT+EXT-models

Figure S7: Interaction of the predictors INT and EXT with age in INT+EXT models.

S-III.3. Model outcome interpretation

While different phonological distance measures employed influenced model fit slightly, they did not influence the direction of the effects of the individual predictors reported. Given that we found considerable differences between the measures (see the correlations reported in Section S-I.1), this is, to some extent, a surprising result. As already mentioned in Section S-II, the normalization we used may have impacted whether two words are assumed to be connected in the networks. To circumvent the impact of the normalization, additional models defined different thresholds according to which words are assumed to be connected in the networks, e.g., assuming all word pairs in the 0.25 quartile of the non-normalized pairwise distance measures to be connected. The outcomes of these additional models are very similar to those reported above and can be found on the OSF page of this project (link above).

While these results are reassuring with regard to the impact of the different predictor variables on vocabulary growth, there is less clarity on the influence of the different phonological distance measures on model fit, and consequently predicting vocabulary development. Across analyses, the FDMS models, i.e., models using the FDMS phonological distance measure, were best able to capture the data. This might be taken to support the superiority of this measure in terms of capturing how similar two words sound. With the caveat that this is but one interpretation of this finding, there is a need for studies examining the psychological reality of the perceived similarity of words judged to be similar according to FDMS, and indeed, the other measures suggested. Should measures of perceived similarity align with the FDMS scores, this raises interesting questions for the factors that contribute to the perceived similarity of two words. Thus, for instance, the difference between FDL and FDMS centered around the inclusion of vowel similarity scores as well as the comparison of the phonological similarity of syllables in words regardless of the position of the syllable within a word in FDMS. To the extent that these factors then contribute to the perceived similarity of words, this would suggest that they are relevant for lexical processing. Furthermore, we note that better insights into phonological similarity of words could also consider more detailed information about words. To avoid the use of normalization and thresholds, one could use weighted edges between nodes containing information about the strength of connections between words, i.e., if words are extremely similar in phonology or just moderately similar, and the extent to which the strength of connections influences the pattern of vocabulary growth. At the very least, the results of the current study suggest that previously reported differences in the influence of INT and EXT growth scenarios on vocabulary development cannot be attributed to the different phonological measures used.

S-IV. Individual effect sizes of INT and EXT per child

At the request of a reviewer, we fitted an additional model that allows individual differences for INT and EXT by child (produced ∼(INT + EXT) * (mom_ed + birth_order + age) + length + frequency + sex + (1 + INT + EXT + frequency || child) + (1 + frequency + age || category)). The results (Figure S8) show that, depending on the child, the networks follow INT growth more strongly and sometimes EXT growth. For most children, INT predominates (green dots

in the scatterplot), but for some children INT and EXT are equally influential (blue dots in the scatterplot), and for some other children EXT predominates. In general, INT and EXT are also anti-correlated – when the influence of INT increases, the influence of EXT decreases and vice versa. This extends to the point that INT, which usually has a positive influence on learning, can also have a negative effect on word learning when EXT influence is high. In the same way, EXT can also have a negative effect. However, most networks show a positive influence of INT and EXT.

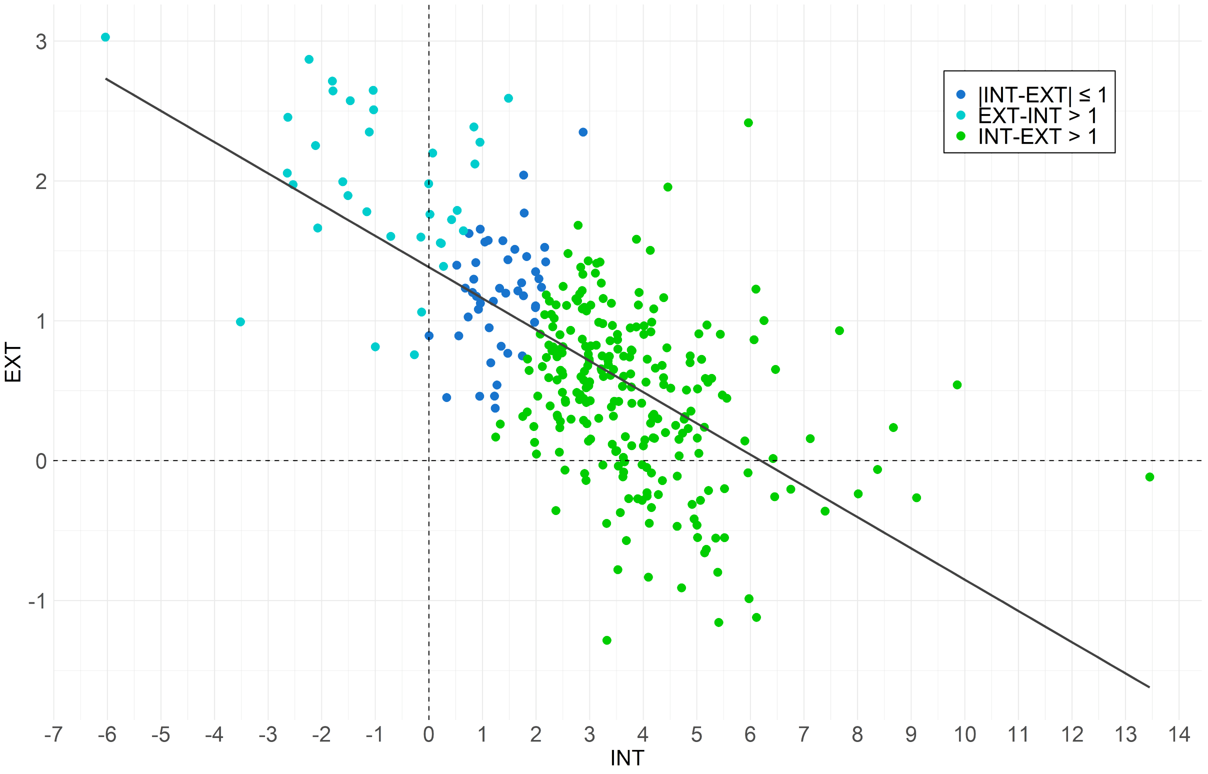

Figure S8: Impact of INT and EXT for each individual child (represented by a dot). The impact was calculated by combining fixed and random effects: for each child, the fixed effect estimate was added to the random effect. This provides the total effect for each individual child, which includes the average effect (fixed) and the individual deviation (random).

Therefore, we can conclude that the networks of most children grow following INT and EXT mechanisms, with INT having a stronger effect in most cases; however, in some cases the vocabularies grow in INT-ways while EXT has a negative influence, or in EXT-ways with a negative influence of INT, respectively. The children's vocabulary networks therefore do not all grow in the same way, with some prioritizing the phonological similarity of words in their environment, but most the similarities in their lexicon.

S-V. Number of words produced per child at first observation

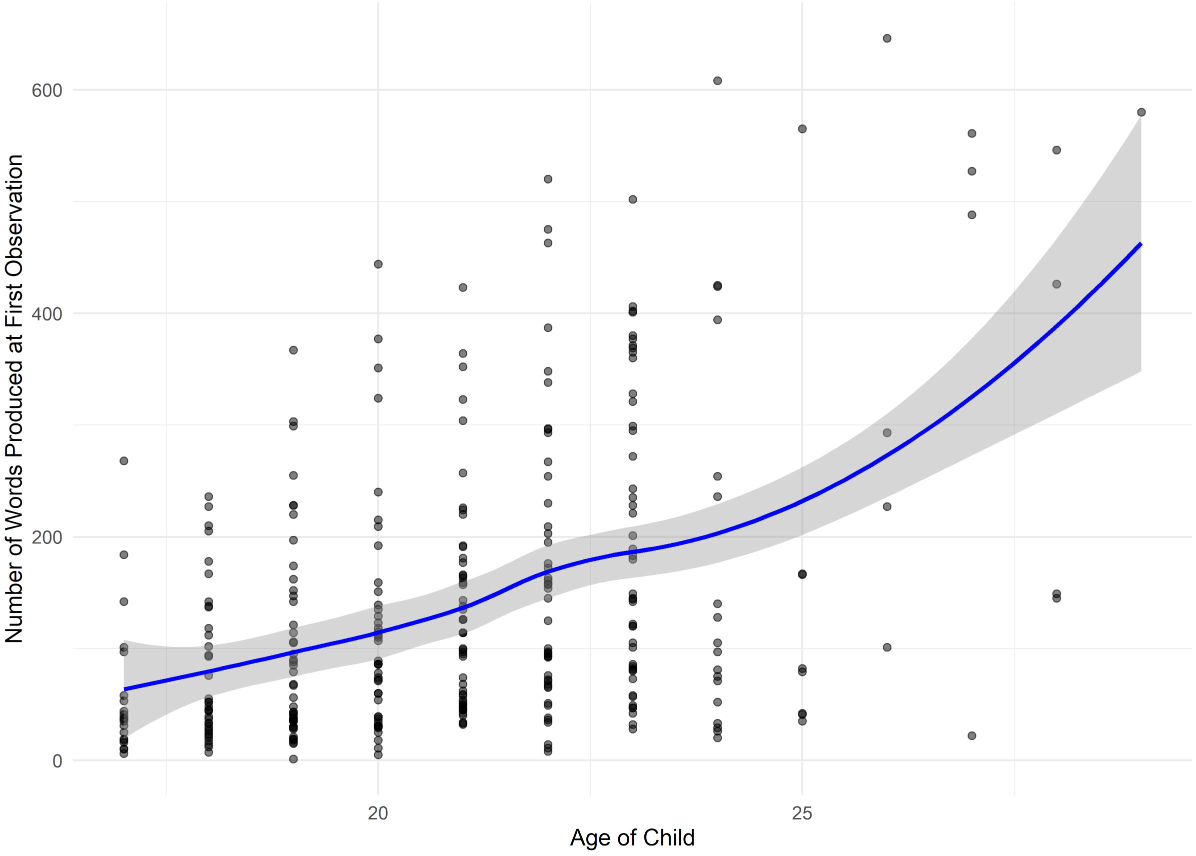

Figure S9: Number of words produced per child at first observation in the CDI.

References

Fourtassi, A., Bian, Y., & Frank, M. C. (2020). The growth of children's semantic and phonological networks: Insight from 10 languages. *Cognitive Science*, *44*(7), e12847. doi: 10.1111/cogs.12847

Harm, M. W., & Seidenberg, M. S. (1999). Phonology, reading acquisition, and dyslexia: insights from connectionist models. *Psychological review*, *106*(3), 491. 10.1037/0033-295X.106.3.491.

Heeringa, W. J. (2004). *Measuring dialect pronunciation differences using levenshtein distance* (Doctoral dissertation, University of Groningen). https://pure.rug.nl/ws/portalfiles/portal/9800656/thesis.pdf.

Kristoffersen, G. (2000). *The phonology of norwegian.* OUP Oxford. doi: 10.1093/oso/9780198237655.001.0001

Laing, C. (2024). Phonological networks and systematicity in early lexical acquisition. *Journal of Experimental Psychology: Learning, Memory, and Cognition.*

Monaghan, P., Christiansen, M. H., Farmer, T. A., & Fitneva, S. A. (2010). Measures of phonological typicality: Robust coherence and psychological validity. *The Mental Lexicon*, *5*(3), 281–299. doi: 10.1075/ml.5.3.02mon

Schepens, J., Dijkstra, T., & Grootjen, F. (2012). Distributions of cognates in europe as based on levenshtein distance. *Bilingualism: Language and Cognition*, *15*(1), 157–166. doi: 10.1017/S1366728910000623

Simonsen, H. G., Kristoffersen, K. E., Bleses, D., Wehberg, S., & Jørgensen, R. N. (2014). The norwegian communicative development inventories: Reliability, main developmental trends and gender differences. *First language*, *34*(1), 3–23. doi: 10.1177/0142723713510997
